# Long-term efficacy and stability of miniscrew-assisted rapid palatal expansion in mid to late adolescents and adults: a systematic review and meta-analysis

**DOI:** 10.1186/s12903-023-03574-y

**Published:** 2023-11-03

**Authors:** Weiqian Zeng, Shuyun Yan, Yating Yi, Hao Chen, Tongke Sun, Yimeng Zhang, Jun Zhang

**Affiliations:** 1 Department of Orthodontics, School and Hospital of Stomatology, Cheeloo College of Medicine, Shandong University & Shandong Key Laboratory of Oral Tissue Regeneration & Shandong Engineering Research Center of Dental Materials and Oral Tissue Regeneration & Shandong Provincial Clinical Research Center for Oral Diseases, No.44-1 Wenhua Road West, 250012 Jinan, Shandong China; 2 The Second Affiliated Hospital of Shandong University of Traditional Chinese Medicine, Jinan, Shandong China

**Keywords:** MARPE, Miniscrew, Palatal expansion, Efficacy, Stability, Late adolescents, Adults

## Abstract

**Background:**

The purpose of this study is to investigate the long-term efficacy and stability of Miniscrew-assisted Rapid Palatal Expansion (MARPE), including its primary outcomes, namely the nasomaxillary complex transverse skeletal and dental expansion, and related secondary outcomes.

**Methods:**

Electronic databases and manual literature searches, up to October 31, 2022, were performed. The eligibility criteria were the following: studies on patients with transverse maxillary deficiency treated with MARPE in adults and adolescents over 13.5 years of age.

**Results:**

Ultimately, twelve articles were included in the analysis, one prospective and eleven retrospective observational studies. Five studies showed a moderate risk of bias, while the remaining seven studies were at a serious risk of bias. The GRADE quality of evidence was very low. MARPE is an effective treatment modality for transverse maxillary deficiency (mean success rate: 93.87%). Patients showed increased mean in the skeletal and dental transverse expansion. The basal bone composition, mean alveolar bone and mean dental expansion accounted for 48.85, 7.52, and 43.63% of the total expansion, respectively. There was a certain degree of skeletal and dental relapse over time. MARPE could also cause dental, alveolar, and periodontal side effects, and have an impact on other craniofacial bones, upper airway, and facial soft tissue.

**Conclusions:**

MARPE is an effective treatment for transverse maxillary deficiency, with a high success rate and a certain degree of skeletal and dental relapse over time.

**Supplementary Information:**

The online version contains supplementary material available at 10.1186/s12903-023-03574-y.

## Background

Transverse maxillary deficiency is considered a relatively common orthodontic problem [1, 2], reported with an incidence rate of 7.9% in adolescents and 10% in adults [3]. It is usually associated with unilateral or bilateral posterior crossbite, dental crowding, deep and narrow palate, vertical alveolar overgrowth, large buccal corridors, facial muscular imbalance, as well as nasal stenosis and airway stenosis [4–7]. It is necessary for orthodontists to establish a normal transverse skeletal relationship between the upper and lower jaws.

Optimal timing of treatment is critical to correct transverse discrepancy of the maxilla [8], since its success is related to mid-palatal suture (MPS) fusion. Rapid maxillary expansion (RME) has proven to be a conventional and widely accepted method to correct transverse maxillary deficiency before the peak of skeletal growth [9], which can be used to widen the width of the maxilla by applying a transverse force to the maxillary teeth, in order to effectively open the palatal suture of children and young adolescents. However, as the suture fusion advances, the resistance to suture opening increases [10, 11].

Limitations and adverse effects of conventional RPE in people over 15 years of age are common, such as buccal crown tipping, alveolar bone dehiscence, decrease of thickness and level of the buccal and lingual bone, gingival retraction, root resorption, pain, limited or failed skeletal expansion, and post-expansion relapse [12]. Therefore, surgically-assisted rapid maxillary expansion (SARME), an invasive surgical procedure performed to correct transverse discrepancies in mid to late adolescents and adults with skeletal maturation, is commonly used to overcome the resistance of suture and limitations of side effects [13]. Although SARME is considered a simple, safe and proven procedure, risks inherent in surgery, high costs and various complications, such as epistaxis, postoperative pain, periodontal problems, asymmetry and incorrect expansion, may result in limitations on patients undergoing such procedures [13].

In order to simplify the treatment procedure and reduce the above adverse reactions, orthodontists began to look for more minimally invasive treatments. Thus, their search facilitated the development of the MARPE procedure, which involves the use of a conventional RPE device, rigid elements and miniscrews implanted in the palate [14]. Compared with RPE, MARPE could deliver the expansion force to the maxillary basal bone directly, produce more skeletal effects and minimize unwanted side effects [15]. A recent clinical study reported that MARPE has a high success rate and causes less trauma, thus it is recommended as an alternative method to surgical expansion [16].

MARPE has incomparable advantages in the treatment of transverse maxillary deficiency, so it has attracted wide attention from orthodontists, and some researchers have conducted multi-dimensional research on this treatment modality [17, 18]. Researchers have proposed that the nasomaxillary complex and even the pterygoid bone, zygomatic bones and temporal bones will change with palatal expansion [19]. Previous studies have systematically reviewed the efficacy of MARPE in mid to late adolescents and adults [20]. However, according to the literature reviewed by our group, no systematic review on the long-term evaluation of the efficacy of this procedure has been reported.

Therefore, this study aims to investigate the long-term efficacy and stability of the MARPE procedure, including its primary outcomes, namely the nasomaxillary complex transverse skeletal and dental expansion, and related secondary outcomes, such as the success rate, duration, buccal crown tipping, effects of alveolar bone, periodontal side effects, root resorption, upper airway changes, facial soft tissue effects, pain, post-expansion relapse, and the possible factors that potentially affect post-expansion changes.

## Methods

### Protocol and registration

This systematic review reports follows the Preferred Reporting Items for Systematic Reviews and Meta-Analyses (PRISMA) guidelines [21]. The review protocol was registered at PROSPERO with the registration number CRD42022323832. Detailed information of the protocol can be found on the PROSPERO website.

### Eligibility criteria

According to the research objectives, the eligibility criteria were determined in advance. Studies of treating transverse maxillary deficiency with the MARPE procedure in adults and adolescents over 13.5 years of age, including all types of MARPE appliance designs, were considered eligible. At least one of the primary outcomes should be reported (efficacy and stability of MARPE about the nasomaxillary complex transverse skeletal and dental expansion), or any other secondary outcomes (success rate, duration, buccal crown tipping, effects of alveolar bone, periodontal side effects, root resorption, upper airway changes, facial soft tissue effects, pain, post-expansion relapse, and the possible factors that potentially affected post-expansion changes) should be included. Additionally, randomized clinical trials, non-randomized clinical trials, prospective studies and retrospective studies, were considered eligible.

Studies that included patients under 13.5 years of age, patients with cleft lip or any other craniofacial syndrome diagnosis, patients with a history of maxillofacial surgery, or patients with systemic disease, were excluded. Case reports and in vitro simulations, such as finite element analysis (FEA), were also excluded.

### Information sources and search strategy

A PICOS questionnaire was developed to select search terms more accurately and comprehensively and was as follows. Population: treating transverse maxillary deficiency with MARPE. Intervention: miniscrew-assisted rapid palatal expansion. All types of MARPE device designs were accepted. Control and outcome were not specified, in order to collect literature more extensively.

A comprehensive electronic database search of the literature was performed in the following databases: MEDLINE (via PubMed), Embase, Cochrane Library, Web of Science, Scopus, Chinese National Knowledge Infrastructure (CNKI), and Wanfang. In addition, we searched the "grey" literature through a Google Scholar web search. A search strategy was developed for MEDLINE, and corresponding modifications were made according to other databases. Additionally, a manual search was also performed for the bibliography of selected articles that may have been omitted. There were no language or publication restrictions. All studies published before October 31, 2022 were included in the search. The details of the searches are shown in Supplementary Table 1.

### Study selection, data items and collection

Eligibility assessment was performed independently without blinding by three reviewers. Two reviewers (W. Z. and Y. Y.) screened the titles and abstracts of the retrieved records based on the predetermined eligibility criteria and removed duplicates. Also, full text was accessed to check for eligibility. Any disagreements between reviewers were resolved through discussion with the third reviewer (J.Z.). If the required information was not provided, we would try to contact the corresponding author by e-mail.

The data collection and extraction: titles, study characteristics (authors, publication year, country, journal, and setting), methods (study design, data collection, and measurements), population (sample size, sex, age range, and mean age), intervention (type of MARPE device, miniscrews, device location, expansion protocol, retention and duration) and outcomes (any primary outcomes and secondary outcomes).

### Risk of bias in individual studies and risk of bias across studies

According to the Cochrane Handbook for Systematic Reviews of Interventions [22], the Revised Cochrane Risk of Bias Tool for Randomized Trials (ROB) [23] and the Risk Of Bias in Non-randomized Studies of Interventions (ROBINS-I) tool [24] were used for observational research to assess the risk of bias in the selected studies. Seven components of bias were evaluated with the ROB tool, namely (1) random sequence generation, (2) allocation concealment, (3) blinding of participants and personnel, (4) blinding of outcome assessment, (5) incomplete outcome data, (6) selective reporting, and (7) other bias. An overall assessment of bias (high, unclear, low) was performed for each included study. Ultimately, studies with high risk were excluded from the meta-analysis. Seven components of bias were evaluated in accordance with the ROBINS-I tool, namely (1) bias due to confounding, (2) bias in the selection of participants into the study, (3) bias in the classification of interventions, (4) bias due to deviations from the intended intervention, (5) bias due to deviations from the intended intervention, (6) bias in the measurement of outcomes, and (7) bias in the selection of the reported result. An overall assessment of bias (Low, Moderate, Serious, Critical) was made for each included study. Studies with a risk of critical bias were excluded from further analysis and synthesis. Any differences between the reviewers were resolved through discussion and consensus among all three reviewers.

The response options for an overall risk of bias were obtained based on each evaluation tool.

### Synthesis of results and summary measures

Mean differences (MDs) and corresponding 95% confidence intervals (CIs) were calculated in millimeters for the primary outcomes: maxillary transverse skeletal and dental expansion. To evaluate the heterogeneity among studies, a Q statistic and a I^2^ statistics were calculated to assess heterogeneity. A fixed-effects model was selected when homogeneity was accepted, while a random-effects model was used when homogeneity was rejected (*P*-value of Q statistic < 0.10, or/and I^2^ > 50%). The Stata software version 12.0 (Stata Corporation, College Station, TX, USA) was used for data analysis and synthesis by one author (W.Z.).

### Quality of evidence

The overall quality of the evidence was rated by using the Grading of Recommendations Assessment, Development and Evaluation (GRADE) system [25]. Any differences between the reviewers were discussed and resolved by consensus among all three reviewers.

### Sensitivity analyses and additional analyses

Robustness of the results was evaluated for meta-analyses by sensitivity analysis. Sources of heterogeneity were sought through sensitivity analysis and, if possible, were further sought through subgroup analysis according to age, cervical spine staging (CVS), MPS, type of device, retention and monocortical or bicortical anchorage. Publication bias was assessed with ≥ 8 studies by the Egger's test, and was considered statistically significant when *P* < *0.05*.

## Results

### Study selection and characteristics

**Fig. 1:**
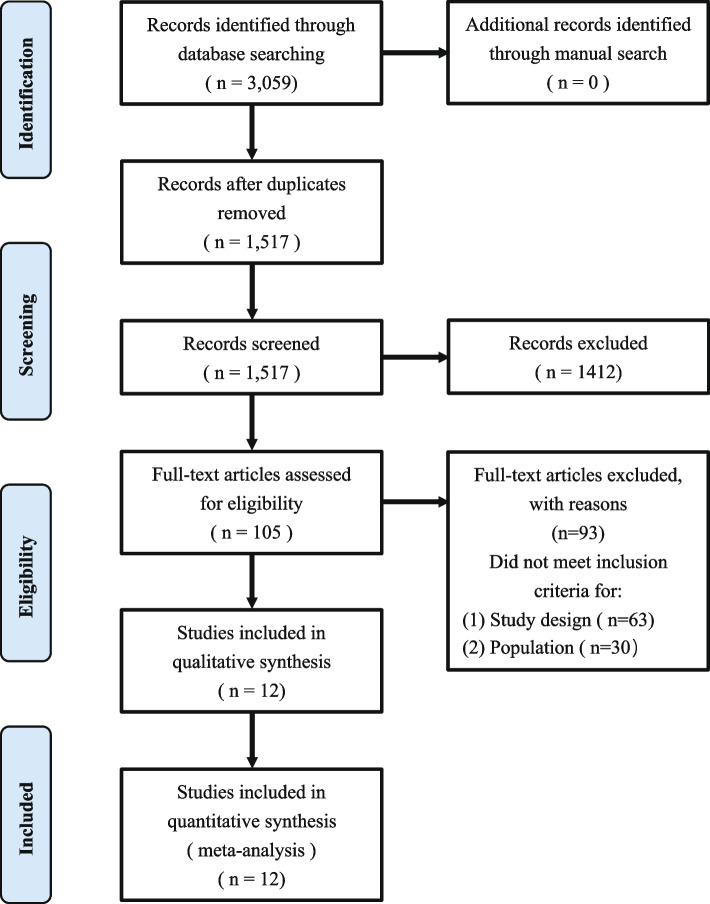
PRISMA flow diagram showing the study's screening and selection process

The electronic database search process is illustrated in Fig. 1. A total of 3,059 studies were retrieved through database searching (MEDLINE *N* = 527, Embase *N* = 449, Cochrane Library *N* = 373, Web of Science *N* = 786, Scopus *N* = 830, CNKI *N* = 51, Wanfang *N* = 43) and no new articles were included through manual search. After removal of duplicates, 1,517 studies were screened based on title and abstract and 105 studies were selected for full text screening (Supplementary Table 2: exclusions). Ultimately, 12 articles were included, and the main characteristics are summarized and reported in Table 1. Most of the literature was excluded, as the follow-up time did not meet the preset criteria of this review or included patients under 13.5 years of age.

**Table 1:** Characteristics of included studies

Study& year	Setting& country	study design	Sample size	Sex	Age (range, mean ± SD)	Data collection	Intervention: appliance type, location, tads	Intervention: expansion protocol	Retention	Duration of expansion	Outcomes	Success rate: (N, %)
Winsauer et al. 2021 [26]	Academic, Austria	Retrospective study	*N* = 32	Male = 8 (29.6%) Female = 19 (70.4%)	18– 49 years 26.8 ± 8.2 years	CBCT images at T1: before treatment (range, 1–7 days) T2: after expansion (range, 2–4 months)	MICRO-4 device (fabricated in the laboratory) with 4 miniscrews (Dual Top Jetscrew, Jeil Medical, South Korea) Anterior position (at P1) Middle position (at P2) *N* = 4, L: 14–16 mm, D: 2.5 mm	2-stage protocol: 1. activation period: the device was activated for 1 week (2 times/d, 1/6 turn/times, 0.34 mm/d) 2. forced controlled polycyclic expansion period: Every third day, the device was additionally activated by 0.17 mm again until the desired maxilla expansion was reached	MICRO-4 device for about 9 months and a mini screw borne transpalatal arch for another 12–15 months	Duration of expansion 81.2 ± 31.0 days	1. Success rate 2. Complication rate: -Dental (gingival irritation, increased periodontal probing depth, root resorption or damage, gingival recession, loss of vitality) -Tissue (peri-implantitis, infection, ulceration) -Hardware-related side effects (loosening or deformation of miniscrew or abutment, fracture or deformation of expansion screw) -Anatomical complications (asymmetric expansion, fracture of bone)	27/32 84.4%
An et al. 2021 [27]	Academic, Korea	Retrospective study	*N* = 21	Male = 3 (14.3%) Female = 18 (85.7%)	at least 18 years 21.97 ± 6.49 years	Posteroanterior and lateral cephalograms and frontal photographs at T1: pretreatment T2: after expansion (at least 6 weeks after cessation of expansion, post-expansion duration 2.91 ± 0.59 months)	MSE type I appliance (Biomaterials Korea, Inc., Seoul, Korea) with bands on P1s and M1s Anterior position: at P1 Posterior position: at M1 *N* = 4, L: 11 mm, D: 1.5 mm	Started at 2 weeks after the delivery of the expander, 2 times/d, 1/4 turn/times, (0.4 mm/d), followed by 1/4 turn/d (0.2 mm/d) after midpalatal suture was opened, until the maxillary molar palatal cusp contacted with the lingual inclination of the buccal cusp of the mandibular molar	At least 6 weeks	30.95 ± 13.09 days	1. Skeletal -Facial width: the distance between the Lt&Rt zygia -Nasal width: the longest distance between Lt&Rt lateral bony walls of the nasal cavity -Maxillary width: the distance between Lt&Rt jugal points 2.Dental -Intermolar root width: the distance between Lt&Rt buccal root tips of the maxillary M1 -Intermolar crown width: the distance between the most lateral points on the buccal surfaces of the maxillary M1 crowns	21/21 100%
Li Q et al. 2020 [28]	Academic, China	Retrospective study	*N* = 22	Male = 4 (18.2%) Female = 18 (81.2%)	18–35 years 22.6 ± 4.5 years	CBCT images at T1: before treatment T2: 3 months after expansion	MSE type II appliance (BioMaterials Korea, Seoul, Korea) with bands on M1s Posterior position: at M1 *N* = 4, L: 11 mm, D: 1.5 mm	Immediate expander activation (4 turns), followed by 2 turns/d (one turn, ¼, 0.13 mm) until maxillary skeletal width was no longer less than that of the mandible	3 months	30–43 days mean 38 days	1. Vertical and horizontal dimensions and volume of the nasal cavity, nasopharyngeal, retropalatal, retroglossal and hypopharyngeal airway 2. Skeletal -Nasal osseous width: Nasal lateral width, Nasal lateral width -Maxillary width: tangent to the NF, tangent to HP	22/22 100%
Yi et al. 2020 [29]	Academic, China	Retrospective study	Total *N* = 35 Included *N* = 19	Male = 4 (21.1%) Female = 15 (78.9%)	15–29 years 19.95 ± 4.39 years	CBCT images at Initial diagnosis 3 months after MARPE treatment	MARME appliance with miniscrews (Ormoc VectorTAS) Anterior position: between C and P1 Posterior position: between P2 and M1 N = 4, L: 8 mm (12 mm), D: 1.4 mm (1.6 mm)	2 times/d (0.25 mm/turn/times, interval 12 h) for 14 days until expansion was achieved 7 mm			1. Skeletal Maxillary width (P1, P2, M1, M2): tangent to NF, tangent to HP, 5 mm below HP, midpalatal width, suture 2. Dental Distance between buccal cusps: P1, P2, M1, M2 3. Upper airway: volume, area, length	29/35 82.9%
Li N et al. 2020 [30]	Academic, China	Retrospective study	Total N = 48 G1: 4-all-bicortical, N = 17 G2: 2-rear-bicortical, N = 17 G3: non-4-bicortical, N = 14	Male = 20 (39.6%) Female = 28 (60.4%) G1: Male = 7 (41.2%) Female = 10 (58.8%) G2: Male = 8 (47.1%) Female = 9 (52.9%) G3: Male = 5 (35.7%) Female = 9 (64.3%)	15–26 years 19.4 ± 3.3 years G1: 15.1–24.5 years 19.5 ± 3.1 years G2: 15.5–25.6 years 19.2 ± 3.5 years G3: 15.7–24.8 years 19.6 ± 3.5 years	CBCT images at before treatment 3 months after activation (3.4–4.9 months)	MSE type II (BioMaterials Korea, Seoul, South Korea) with bands on M1s Posterior position: at M1 *N* = 4, L: 11 mm, D: 1.5 mm	1/6 turn/d (0.13 mm/d) until the maxillary skeletal width was no longer less than that of the mandible	3 months		1. Skeletal -Nasal width: most lateral wall of the nasal cavity -Maxillary width: tangent to NF (M1), parallel to HP (M1), 5 mm above NF, 5 mm below HP -Nasomaxillary width: lateral pterygoid, zygomatic bone, temporal bone 2. Alveolar bone -Inclination: M1 -Alveolar bone loss: alveolar crest on the buccal side, M1 3. Dental -IMW (tooth apices, central fossae) -Tooth inclination: M1	48/48 100%
Lin et al. 2015 [15]	Academic, Korea	Retrospective study	*N* = 15	Female = 15 (100%)	18.1 ± 4.4 years	CBCT images at T1: before treatment T2: 3 months after activation	C-expander, supported with 4 TSADs (Complaint Co, Seoul, Korea), placed 8 mm beneath the alveolar ridge on the palatal slope Anterior position: between C and P1 Posterior position: between P2 and M1 *N* = 4, L: 8.5 mm, D: 1.8 mm	Activated over 7 mm after placement, followed by 1/4 turn/d (0.25 mm/d)			All measurements were performed on Ps and Ms palatal suture (x-plane), parallel to the palatal plane (y-plane), tangent to NF (z-plane) 1. Skeletal -Midpalatal suture -NF, HP, and 5 mm below HP 2. Alveolar -Inclination -Buccal dehiscence 3. Dental -Tooth apex and crown level -Inclination -Vertical height of tooth	15/15 100%
Alsayegh et al. 2022 [31]	Academic, UAE	Retrospective study	*N* = 28	Male = 19 (67.9%) Female = 9 (32.1%)	at least 16 years, mean age of 20.9 years	Digital STL models utilizing 3Shape Ortho Analyzer 3D scanner software at before treatment 3 months after activation Interval = 24.1 ± 9.3 months (active orthodontic treatment time)	MARPE appliance with miniscrews (ORLUS, Ortholution, Seoul, Korea) *N* = 4, L: 7 mm, D: 1.8 mm	a quarter of a turn (0.2 mm) every second day until the palatal cusp of the maxillary first molars came in contact with the buccal cusp tips of the mandibular first molars	3 months		Dental -ICW, IMW -Inclination:M1	28/28 100%
McMullen et al. 2022 [32]	Academic, USA	Retrospective study	*N* = 14	Male = 6 (42.9%) Female = 8 (57.1%)	19.9 ± 4.8 years	CBCT images at T1: before treatment T2: after the maxillary expander removal; 6 months after the initial CBCT scan Interval = 6.0 ± 4.3 months	MSE appliance (a central expansion jackscrew with 4 attached arms soldered to orthodontic bands placed on maxillary M1. The addition of 4 sheaths welded to the body of the central expansion jack screw allowed for the placement of the miniscrews in the roof of the mouth) Position: posteriorly without extending into the palatine processes *N* = 4, L: 8–12 mm, D: 1.8 mm	Activation protocol began 2 weeks after the placement of the miniscrews. The rate of activation was standardized according to the subjects’ chronological age. 13-15y, initial 2 turns/d, after opening of the diastema 2 turns/d 16-17y, initial 2–3 turns/d, after opening of the diastema 2 turns/d 18y, initial 3–4 turns/d, after opening of the diastema 2–3 turns/d The expansion was concluded when the lingual cusp of the maxillary molar contacted the tip of the mandibular molar buccal cusp. If the expansion occurred asymmetrically, it was stopped according to the side that expanded more			1. Maxillary lateral displacements (Difference between T2—T1 measurements): Distance between Lt&Rt Or, Distance between Lt&Rt zygomatic, Distance between Lt&Rt nasal cavity, Distance between Lt&Rt PF, Distance between Lt&Rt canine cusp tip, Distance between Lt&Rt molar cusp tip 2. Maxillary anteroposterior, superior-inferior, and 3D displacements (Midpoints were generated for each bilateral landmark, and then the difference was taken from T1—T2): Or midpoint, Zygomatic midpoint, Nasal cavity midpoint PF midpoint, Canine cusp tip midpoint, Molar cusp tip midpoint, Anterior nasal spine (ANS, T2 only), Posterior nasal spine (PNS, T2 only), A-point (T2 only) 3. Angular Changes -Palatine plane: anterior and posterior nasal spine (ANS-PNS), Angle formed by the Lt&Rt Or-zygomatic lines in the anterior view -Molar torque: long axis of the molars -Canine torque: long axis of the canines	14/14 100%
Calil et al. 2021 [33]	Academic, Brazil	Retrospective study	N = 16	Male = 5 (31.3%) Female = 11 (68.8%)	at least 16 years 24.92 ± 7.60 years	CBCT images at T1: before treatment T2: after expansion Interval = 6 months	MARPE technique with the PecLab appliance (Belo Horizonte, Minas Gerais, Brazil) with bands on M1s Posterior position: at M1 *N* = 4, L: 8 mm, D: 1.8 mm	2/4 turn/d until the palatal cusps of the maxillary first molars touch the buccal cusps of the mandibular first molars	4 months		1. Skeletal: nasal base width and jugula width 2. Dental: -ICW, IP1W, IP2W, IMW -Inclinations (C, P1, P2, M1): measured by the line passing through the long axis of the tooth and vertical line parallel to the midsagittal plane 3. Alveolar: -Buccal bone thickness (at M1s, mesiobuccal and distobuccal roots; P1s, buccal; P2s, buccal, C, buccal) in axial slices, 4 mm above the cementoenamel junction in the mesial of the Rt M1	16/16 100%
Tang et al. 2021 [19]	Academic, China	Retrospective study	Total *N* = 31 Included *N* = 19	Male = 12 (38.7%) Female = 19 (61.3%)	18–33 years 22.14 ± 4.76 years	CBCT images at T0: before treatment T1: after retention T2: after debonding Interval: T1–T0: 6 ± 1.9 months T2–T1: 13 ± 2.18 months	MSE type II appliance (BioMaterials Korea, Seoul, Korea) with bands on M1s Posterior position: at M1 *N* = 4, L: 11 mm, Insertion depth = 8.7 mm, D: 1.5 mm	1 turn/d (0.13 mm/turn) According to the amount of maxillary width deficiency of each patient ranging from 40–60 turns	At least 3 months, followed by passive retention (jackscrew and four mini-implants were kept until the brackets were debonded)	40–60 days	Skeletal -Maxillary width: -tangent to the NF at most inferior level, tangent to HP, 5 mm below HP -Nasal width: nasal lateral width -Posterior midpalatal suture width -Palatal bone thickness -Palatal cortical bone thickness -Distance between the Lt&Rt lateral pterygoid plate -Distance between the foramina of the Lt&Rt zygomatic bones -Distance between the Lt&Rt temporal bone	28/31 90.3%
Lim et al. 2017 [34]	Academic, Korea	Retrospective study	Total *N* = 38 Included N = 24	Male = 8 (33.3%) Female = 16 (66.7%)	18.25–26.75 years 21.55 ± 3.14 years	CBCT images at T0: before (T0) T1: immediately after (within 1 month after the completion of expansion, mean 9.5 days, range 0–28 days) T2: 1 year after the completion of expansion (14.17 ± 2.70 months, range 12.0–16.5 months)	Modified Hyrax II type expander (Dentaurum, Ispringen, Germany) with bands on P1s and M1s Anterior position: 2 in the rugae area Posterior position: 2 in the para-midsagittal area at M1 *N* = 4, L: 7 mm, D: 1.8 mm	1 turn/d (0.2 mm/turn) until the required expansion had been achieved	4 months, 15.29 ± 3.05 weeks	5 weeks	1. Appliance expansion 2. Skeletal -Nasal width: nasal cavity, NF (M1) 3. Alveolar -Alveolar width -Alveolar bone inclination: M1 -Alveolar crest levels: interproximal and buccal -Bone thicknesses: buccal and palatal, P1, P2, M1 4. Dental -Intercusp and interapex width: ICW, IP1W, IP2W, IMW -Inclination:M1	33/38 86.8%
Clement et al. 2017 [35]	Academic, India	Prospective study	*N* = 10	Male = 5 (50.0%) Female = 5 (50.0%)	19–24 years mean age of 21.5 years	CBCT, models, and photographs at T1: before insertion of expansion device T2: after stabilization Interval = more than 4 months	MSE (BioMaterials Korea, Seoul, South Korea) with bands on M1s Anterior position: between C and P1 Posterior position: between P2 and M1 *N* = 4, L: 11 mm, D: 1.8 mm	Maxillary expansion was initiated 2 days after insertion of the device. The appliance was then activated 2 turns/day until the required expansion was achieved	4 months		All measurements were performed on Cs, P1s, P2s, M1s the Frankfort horizontal plane (x-plane), the transporionic plane (y-plane), the midsagittal plane (z-plane) 1. Skeletal -Midpalatal suture -Nasal cavity, zygoma, frontonasal level 2. Alveolar -Alveolar width: Medial limits of the alveolar process at Lt&Rt 3. Dental -ICW, IP1W, IP2W, IMW -Inclination	10/10 100%

*C* canine, *P1* first premolar, *P2* second premolar, *M1* first molar, *M2* second molar, *ICW* intercanine width, *IP1W* interpremolar width at P1, *IP2W* interpremolar width at P2, *IMW* intermolar width, *NF* nasal floor, *HP* hard palate, *TAD* temporary anchorage device = miniscrew, *L* length, *D* diameter, *Rt* right, *Lt* left, *d* day, *G* group

Among the 12 included studies, 1 was a prospective observational study and the remaining 11 were retrospective observational studies [15, 19, 26–35].

### Risk of bias within studies

**Fig. 2:**
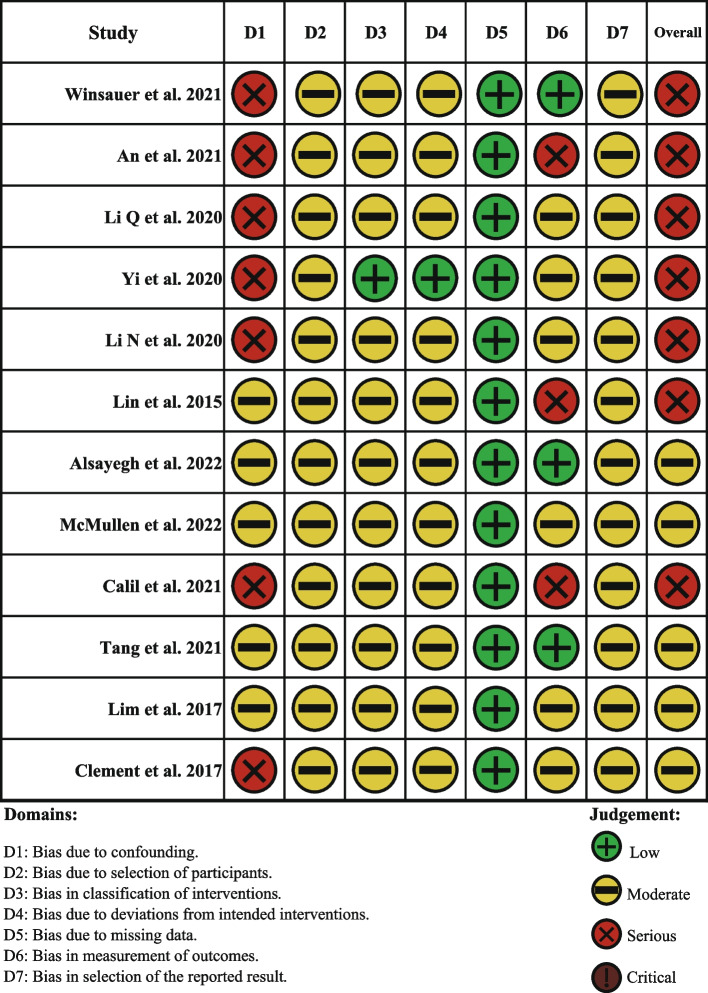
Results of the risk of bias assessment in the individual studies with the ROBINS-I tool

**Fig. 3:**
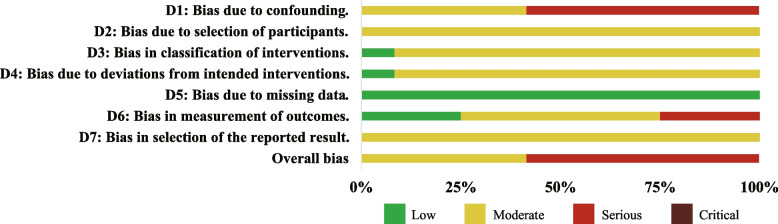
Risk of bias percentage per domain of all included studies assessed with the ROBINS-I tool

Since all of the included studies were observational studies, the ROBINS-I tool was used to assess the risk of bias (Figs. 2 and 3). Five studies showed a moderate risk of bias [19, 31, 32, 34, 35], while the rest seven studies were at a serious risk of bias [15, 26–30, 33]. The main risk of bias comes from bias due to confounding, selection of participants, measurement of outcomes, and selection of the reported results. Although some researchers have recognized that a more scientific approach to maxillary transverse expansion based on the state of the MPS fusion is more appropriate, the vast majority of studies were still grouped according to age, resulting in a confounding in most of the literature included in this review. As for selection bias, the authors used the appropriate methods to adjust for the selection bias, even though the start of the follow up and the start of the intervention do not coincide for all participants. Thus, bias due to selection of the participants was found to be moderate. Regarding the measurement of outcomes, since the device was not removed in some of the studies when the cone beam computed tomography (CBCT) was taken, there was no guarantee that the measurer was not affected by the intervention. Also, there was no clear evidence (such as a pre-registered protocol) that outcome measurements and analyses were consistent with an a priori plan. Additionally, there was no indication of selection of the reported analysis from among multiple analyses and selection of the cohort or subgroups for analysis and reporting on the basis of the results.

### Results of individual studies and meta-analysis

The results of all individual studies for the primary outcomes are summarized in Tables 2 and 3, and the secondary outcomes are included in Supplementary Tables 3, 4 and 5.

**Table 2:** Results of individual studies for skeletal maxillary expansion (at M1) by MARPE. Measurement, mean ± SD (mm), 95% CI, range (mm), *p*-value and effect size were described when available

Measurement	Study	Mean ± SD (mm)	95% CI lower/upper	Range (mm)	*P* value	Effect size
Maxillary basal bone width	Li Q et al. 2020 [28]	2.0 ± 1.0			< .001	
	Yi et al. 2020 [29]	1.67 ± 1.17			0.000	
	Li N et al. 2020 [30]	G1: 4.6 ± 1.2 G2: 4.3 ± 1.0 G3: 3.2 ± 1.1			G1: 0.000 G2: 0.000 G3: 0.000	
	Lin et al. 2015 [15]	1.99 ± 1.18			.0000	
	Tang et al. 2021 [19]	2.23 ± 1.08			< .001	
Maxillary alveolar bone width	An et al. 2021 [27]	2.79 ± 1.59			< 0.001	
	Yi et al. 2020 [29]	1.76 ± 1.19			0.000	
	Li N et al. 2020 [30]	G1: 6.8 ± 1.3 G2: 6.9 ± 1.1 G3: 7.2 ± 1.4			G1: 0.000 G2: 0.000 G3: 0.000	
	Lin et al. 2015 [15]	2.38 ± 1.17			.0000	
	Calil et al. 2021 [33]	3.06 ± 1.81			0.000	
	Tang et al. 2021 [19]	2.56 ± 1.46			< .001	
	Lim et al. 2017 [34]	2.10 ± 1.13			< 0.001	
Nasal floor width	Li Q et al. 2020 [28]	2.3 ± 1.2			< .001	
	Yi et al. 2020 [29]	1.77 ± 1.48			0.000	
	Li N et al. 2020 [30]	G1: 4.2 ± 1.2 G2: 4.0 ± 1.1 G3: 2.3 ± 1.1			G1: 0.000 G2: 0.000 G3: 0.000	
	Lin et al. 2015 [15]	1.87 ± 1.13			.0000	
	Calil et al. 2021 [33]	2.82 ± 1.54			0.000	
	Lim et al. 2017 [34]	1.56 ± 1.02			< 0.001	
Nasal lateral width	Li Q et al. 2020 [28]	2.3 ± 1.2			< .001	
	Yi et al. 2020 [29]	1.54 ± 1.03			0.000	
	Li N et al. 2020 [30]	G1: 3.3 ± 1.1 G2: 3.0 ± 1.2 G3: 2.1 ± 1.0			G1: 0.000 G2: 0.000 G3: 0.000	
	Tang et al. 2021 [19]	2.12 ± 1.08			< .001	
	Lim et al. 2017 [34]	1.25 ± 0.80			< .0001	

*M1* first molar, *CI* confidence interval, *SD* standard deviation, *G* group

**Table 3:** Results of individual studies for dental expansion by MARPE. Measurement, mean ± SD (mm), 95% CI, range (mm), *p*-value and effect size were described when available

Measurement	Study	Mean ± SD (mm)	95% CI lower/upper	Range (mm)	*P* value	Effect size
ICW	Alsayegh et al. 2022 [31]	2.3 ± 1.21			≤ 0.001	
	McMullen et al. 2022 [32]	2.7 ± 1.9				
	Calil et al. 2021 [33]	3.04 ± 2.03			0.036	
	Lim et al. 2017 [34]	2.95 ± 2.43			< 0.001	
	Clement et al. 2017 [35]	5.83 ± 1.32			0.000	
IP1W	Yi et al. 2020 [29]	3.00 ± 2.36			0.000	
	Lin et al. 2015 [15]	4.00 ± 1.27			0.0000	
	Calil et al. 2021 [33]	3.81 ± 2.12			0.377	
	Lim et al. 2017 [34]	4.99 ± 2.24			< 0.001	
	Clement et al. 2017 [35]	5.33 ± 1.72			0.043	
IP2W	Yi et al. 2020 [29]	3.61 ± 2.00			0.000	
	Lin et al. 2015 [15]	3.44 ± 1.13			0.0000	
	Calil et al. 2021 [33]	3.44 ± 2.21			0.512	
	Lim et al. 2017 [34]	3.88 ± 2.21			< 0.001	
	Clement et al. 2017 [35]	5.66 ± 1.36			0.000	
IMW	An et al. 2021 [27]	5.32 ± 2.05			< 0.001	
	Yi et al. 2020 [29]	3.92 ± 2.36			0.000	
	Li N et al. 2020 [30]	G1: 6.8 ± 1.3 G2: 6.9 ± 1.1 G3: 7.2 ± 1.4			G1: 0.000 G2: 0.000 G3: 0.000	
	Lin et al. 2015 [15]	3.46 ± 1.06			0.0000	
	Alsayegh et al. 2022 [31]	4.2 ± 1.87			≤ 0.001	
	McMullen et al. 2022 [22]	3.6 ± 2.1				
	Calil et al. 2021 [33]	6.37 ± 1.72			0.000	
	Lim et al. 2017 [34]	3.61 ± 3.22			< 0.001	
	Clement et al. 2017 [35]	7.33 ± 1.96			0.004	

*ICW* intercanine width, *IP1W* interpremolar width at the first premolar, *IP2W* interpremolar width at the second premolar, *IMW* intermolar width, *CI* confidence interval, *SD* standard deviation, *G* group

### Skeletal transverse expansion of the nasomaxillary complex

Six articles reported transverse expansion of the maxillary basal bone, and all were statistically significant [15, 19, 27–30]. One study was not synthesized due to the use of posteroanterior cephalograms for measurements, whose definitions of the landmarks were different from those of the other studies [27]. In addition, a relatively consistent measurement method, that is, manipulated on CBCT images, was used in the remaining five articles [15, 19, 28–30]. The mean expansion of the basal bone ranged from 1.67 to 4.04 mm. The samples of one study were divided into 3 groups according to the pattern of insertion of the miniscrews used, namely 4-all-bicortical penetration, 2-rear-bicortical penetration, and non-4-bicortical penetration [30]. One study had follow-up time points at 6 ± 1.9 months and 13 ± 2.18 months, and the results were statistically significant at both time points. However, the change between these two time points was a statistically significant decreasing trend [19].

Transverse nasal bone expansion was reported in eleven articles [15, 19, 27–30, 32–36], which were all statistically significant, five of which reported lateral wall of the nasal cavity at the first molar (M1) [19, 28–30, 34], with a mean range of 1.25 to 2.9 mm, five of which reported nasal floor width at M1 [15, 28–30, 33, 34], with a mean expansion ranged from 1.56 to 3.50 mm. Another study was not synthesized for the same reasons described earlier [27]. Two articles measured the variation at the widest part of the pear-shaped foramen, and were not synthesized [32, 35]. The aforementioned article, which was based on the pattern of insertion of the miniscrews used in a subgroup study, also reported nasal bone expansion. The results were also statistically significant for studies followed up at two time points, and the change between these two time points was statistically significantly decreased [19] (Table 2).

### Dental transverse expansion

Nine studies reported the changes in dental transverse widths with nasomaxillary complex expansion [15, 27, 29–35]. Five studies included the intercanine width (ICW) [31–35], five reported inter-first premolar width (IP1W) [15, 29, 33–35], five reported inter-second premolar width (IP2W) [15, 29, 33–35], and nine included the intermolar width (IMW) [15, 27, 29–35]. The mean ICW range was 2.30 to 5.83 mm, the mean IP1W range was 3.00 to 5.33 mm, the mean IP1W range was 3.44 to 5.66 mm, and the mean IMW range was 3.46 to 7.33 mm. The measurements were statistically significant in all but two studies [32, 33] (Table 3).

### Success rate of MARPE

All studies reported the success rate of the MARPE treatment, with a mean success rate of 93.87%, which ranged from 82.9 to 100% [15, 19, 26–35]. In particular, eight studies reported a success rate of 100% [15, 27, 28, 30–33, 35] (Table 1).

### Duration of expansion

All studies described the expansion protocol in detail except for one study. The condition for cessation of activation was usually that the required expansion was achieved. Two of them clearly stated that the expansion was concluded when the width of the maxilla was no longer less than the width of the mandible [28, 30], and four of them were terminated when the maxillary molar palatal cusp contacted with the lingual inclination of the buccal cusp of the mandibular molar [27, 31–33]. Five articles [19, 26–28, 34] reported the duration of activation measured in months, weeks or days. To compare the results, the units of duration was converted into days, and the mean duration of expansion protocol ranged from 13 to 122.2 days (Table 1).

### Retention

Eight articles reported the duration of retention after activation [19, 26–28, 30, 33–35], except for one article, which was at least six weeks [27], the duration of the retention for the remaining studies was at least 3 months. The longest retention was the study conducted by Tang et al., in which the jackscrew and four mini-implants were kept in place as a passive retention until the brackets were debonded, after about 3 months of retention [19] (Table 1).

### Dental side effects

For the buccal inclination of the teeth, different studies used different measurement methods, and the average value varies greatly. Six studies reported dental side effects [15, 30–34]. The reports of maxillary first molars were the most common. And the results in two articles were statistically significant [15, 30].

Among all the articles included, only one discussed the root resorption, but Winsauer et al. did not report the occurrence of root resorption, which included 33 cases [26] (Supplementary Table 3).

### Alveolar and periodontal side effects

Alveolar and periodontal side effects were reported in four articles [15, 30, 33, 34]. Three articles reported the buccal inclination of the alveolar bone [15, 30, 34], three articles reported the change of the alveolar crest level [15, 30, 34], and two articles reported the change of the alveolar bone thickness on the buccal side and (or) the palatal side [33, 34]. One article mentioned the periodontal indicators [26]. For the buccal inclination of the alveolar bone, the angle between the palatal alveolar bone and nasal floor or palatal floor is commonly used, with a mean range from 0.4° to 2.26°, and all were statistically significant [15, 30, 34]. A mean decrease in the buccal alveolar crest level at M1 ranged from 0.11 to 0.8 mm [15, 30, 34], which were statistically significant in all but one studies [34]. The mean range of the decrease of the buccal bone thickness was 0.10 to 0.33 mm [33, 34], and all were not statistically significant (Supplementary Table 4a-c).

### Expansion of other craniofacial bones

Five articles reported the changes of other craniofacial bones, such as the sphenoid bone (which usually refers to lateral pterygoid plate), temporal bones and zygomatic bones [19, 27, 30, 32, 35], and different measurement methods were used in these studies. The zygomatic bone was the most reported as having no statistical significance [27, 32, 35]. In addition, change of the orbital point was only reported in one article [32], and its change was not statistically significant. The other reported changes of skull markers were statistically significant (Supplementary Table 5).

### Changes in the upper airway

Two articles reported on the changes in the upper airway [28, 29]. The nasopharyngeal volume significantly increased after MARPE treatment (*P* < *0.05*) [28, 29], with an increase of 8.48% [29]. However, there were no statistically significant changes in the oropharyngeal, palatopharyngeal, glossopharyngeal and airway total volume (all *P* > *0.05*) [29]. The enlarged nasopharyngeal volume was correlated with the increased nasal width at the posterior nasal spine (PNS) plane (*P* < *0.05*) [28]. There were no correlations between the expanded volume and the maxillary width [28]. The volume of the nasal cavity increased significantly (*P* < *0.05*)[28].

### Effects on soft tissue

Only one article reported the changes in facial soft tissue [27]. The measurement was performed using the frontal image, and included interpupillary distance, alar width, nose length, upper lip length, lip chin length, upper lip vermilion, and lower lip vermilion. Only the changes in the alar width and the nose length were statistically significant in the ranges of 1.18 ± 1.52% and 0.98 ± 2.32%, respectively.

### Synthesis of the results and meta-analysis

**Fig. 4:**
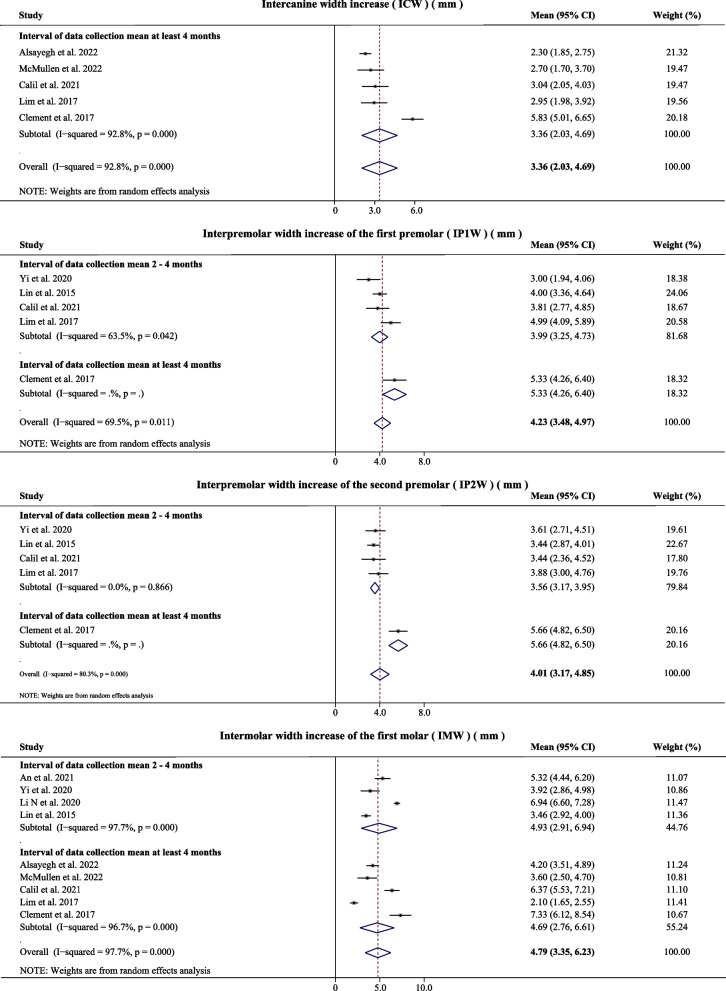
Forest plot of skeletal width increase after MARPE

**Fig. 5:**
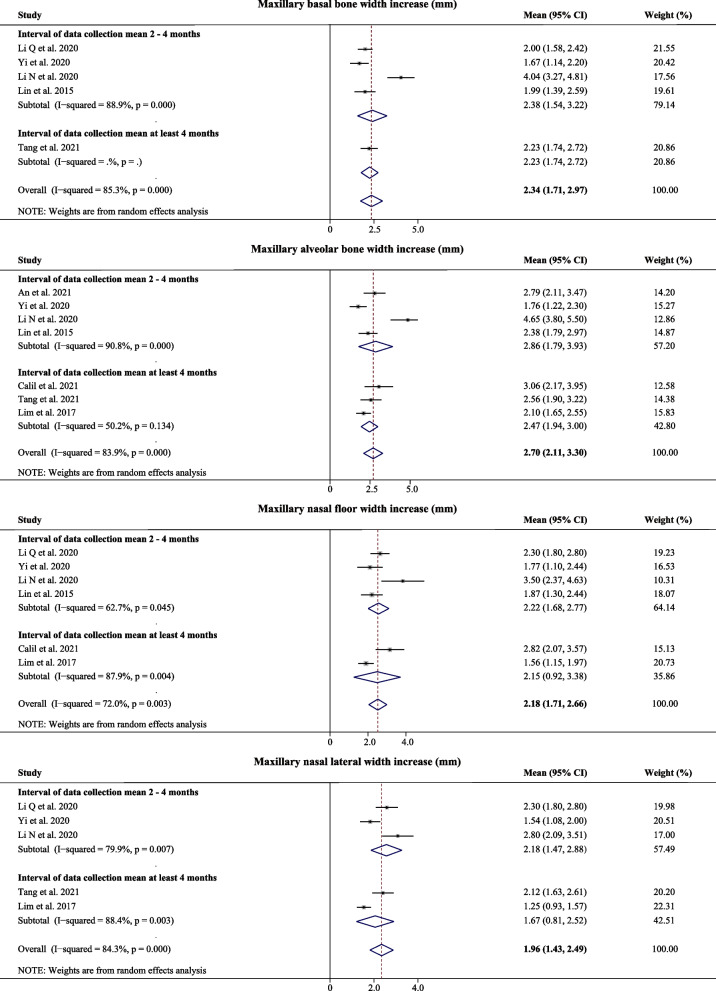
Forest plot of dental width increase after MARPE

There were great differences in methodology among the included studies, such as device design, expansion protocol, measurement and factors that may affect the results. However, the goal of the expansion is to match the width of the maxilla and mandible. The main outcomes were synthesized based on the specified age range and the relatively consistent measurements. The results are shown in Figs. 4 and 5. The increase of the bone width were reported in 8 articles [15, 19, 27–30, 33, 34], including the four indexes, namely the maxillary basal bone width, maxillary alveolar bone width, nasal floor width and nasal lateral width. The dental width was measured at the maxillary canines (C), first premolar (P1), second premolar (P2), and M1 [15, 27, 29–35]. All the synthetic data showed a high heterogeneity (I^2^ > 50%), and a random-effect model was selected. The mean maxillary basal bone width increase was 2.34 mm (95% CI: 1.71–2.97 mm, *P* = *0.000*, I^2^ = 85.3%), the mean maxillary alveolar bone width increase was 2.70 mm (95% CI: 2.11–3.30 mm, *P* = *0.000*, I^2^ = 83.9%), the mean nasal floor width increase was 2.18 mm (95% CI: 1.71–2.66 mm, *P* = *0.003*, I^2^ = 72.0%), and the mean nasal lateral width increase was 1.96 mm (95% CI: 1.43–2.49 mm, *P* = *0.000*, I^2^ = 84.3%), the mean ICW was 3.36 mm (95% CI: 2.03–4.69 mm, *P* = *0.000*, I^2^ = 92.8%), the mean IP1W was 4.23 mm (95% CI: 3.48–4.97 mm, *P* = *0.011*, I^2^ = 69.5%), the mean IP2W was 4.01 mm (95% CI: 3.17–4.85 mm, *P* = *0.000*, I^2^ = 80.3%), and the mean IMW was 4.79 mm (95% CI: 3.35–6.23 mm, *P* = *0.000*, I^2^ = 97.7%).

Since the increase of the IMW represented the combined effect of the maxillary basal bone, maxillary alveolar bone and dental expansion, the mean bone composition accounted for 48.85% of the total expansion, the mean alveolar bone accounted for 7.52% and the mean dental expansion accounted for 43.63% [15, 19, 27–31, 33, 34]. And the mean basal bone composition accounted for 47.55% of the total expansion, the mean alveolar bone accounted for 10.02% and the mean dental expansion accounted for 42.43% in at least four months after expansion.

### Risk of bias across studies and quality of the evidence

Subgroup analysis was performed according to the follow-up time points, including the time points of data collection mean of 2–4 months and at least 4 months, respectively. Except for two outcomes (IP1M, IP2M), the other outcomes showed that the effect of the expansion was worse at the longer follow-up time point, which suggested that the expansion would be accompanied by a degree of relapse. However, no other subgroup analysis was conducted due to the limited eligibility criteria and the inadequacy of the conditions. Sensitivity analyses were performed to search the sources of heterogeneity, but were found to be insignificant.

Egger's test was used to assess the publication bias for the synthetic outcomes that included more than 8 articles. Egger's test for the IMW was evaluated, and no statistical significance was found as all *P-values* were higher than 0.05.

The overall quality of the evidence evaluated by GRADE for the primary outcomes was very low, since observational studies started with a low level and all outcomes downgraded. The main reasons for degradation are risk of bias and inconsistency, and some outcomes showed indirectness and imprecision (Supplementary Table 6).

## Discussion

### Summary of evidence

The aim of the present systematic review and meta-analysis was assessing the long-term efficacy and stability of the MARPE procedure in adults and adolescents over 13.5 years of age. From an initial 3,059 studies, ultimately 12 studies were included according to the eligibility criteria and the assessment of risk of bias [15, 19, 26–35]. Since there were great differences in methodology, device design, expansion protocol, measurement and factors that may affect the results, the main outcomes were synthesized on the basis of the specified age range and the relatively consistent measurements.

Due to the limitations and the common adverse effects of conventional RPE as the suture fusion advances in people over 15 years of age [10, 11], such as buccal crown tipping, alveolar bone dehiscence, decrease of thickness and level of the buccal and lingual bone, gingival retraction, root resorption, pain, limited or failed skeletal expansion and post-expansion relapse [12], it is necessary to determine proper timing for palatal expansion. The interlaced bone spines and "bone islands" begin to appear at the edge of the palatal suture in stage C (2 radiopaque, winding, and parallel lines are separated by areas of low radiographic density). These bone structures will produce the resistance to maxillary transverse expansion, which may be the reason for the poor effect of RME in some patients with stage C [37]. The palatal suture in stages D and E is partially or completely fused, and the resistance is large at this time. Luo reported that the average age for stage C was 13.55 ± 3.29 years [37], and Tonello et al. reported that stage D was more prevalent in 14- and 15-year-olds [38]. Therefore, the present systematic review limited the eligibility criteria to adults and adolescents over 13.5 years of age.

In all studies, an expander with four miniscrews was implanted into the hard palate. There were usually slight differences in the type of miniscrews, among which those with a length of 11 mm and a diameter of 1.5 or 1.8 mm were the most common. The design of the device and the orientation of the jackscrew were similar but different [15, 19, 26–35]. Li N et al. reported that the maxillary skeletal expansion (MSE) with non-4-bicortical penetration produced fewer orthopedic effects and more unwanted dentoalveolar side effects, whereas MSE with 2-rear-bicortical and 4-all-bicortical penetration showed similar skeletal effects, which means that 2-rear-bicortical penetrating miniscrews were necessary for skeletal expansion [30]. This finding suggests that orthodontists should pay attention to the length of the miniscrew and the depth of implantation.

The success of the expansion was defined slightly different. The condition for cessation of activation was usually that the required expansion was achieved. The expansion was generally considered sufficient when the width of the maxilla was no longer less than the width of the mandible [28, 30], or the maxillary molar palatal cusp contacted with the lingual inclination of the buccal cusp of the mandibular molar [27, 31–33]. Five articles [19, 26–28, 34] reported the duration of activation, which ranged from 13 to 122.2 days. The duration of the retention after activation was reported in nine articles [19, 26–28, 30, 31, 33–35]. The duration of the retention was usually at least three months, except for one article (at least six weeks) [27]. The longest retention was reported in the study conducted by Tang et al. in which the jackscrew and four mini-implants were kept in place as a passive retention until the brackets were debonded, after about 3 months of retention [19].

The MARPE procedure proved to be a successful treatment for transverse maxillary deficiency, with a mean success rate of 93.87% [15, 19, 26–30, 32–35], which is consistent with previous studies [20].

Skeletal transverse expansion of the nasomaxillary complex was synthesized in four aspects: maxillary basal bone width, maxillary alveolar bone width, nasal floor width and nasal lateral width. The increase of the mean maxillary basal bone width was 2.34 mm (1.71–2.97 mm), the increase of the mean maxillary alveolar bone width was 2.70 mm (2.11–3.30 mm), the increase of the mean nasal floor width was 2.18 mm (1.71–2.66 mm), and the increase of the mean nasal lateral width was 1.96 mm (1.43–2.49 mm). The maxillary basal bone was closest to the jackscrew, which can best represent the expansion of the palatal suture. The increase of the maxillary alveolar bone width was greater than that of the maxillary basal bone width, while the increase of the nasal floor width and nasal lateral width were in turn smaller. This suggests that as other cranial bones and their suture attachments have not been change, the skeletal transverse maxillary expansion is embodied in "A" type.

According to the meta-analysis published by Kapetanović et al. the mean skeletal expansion was 2.33 mm (1.63–3.03 mm) immediately after expansion [20], which is consistent with the results of this study. However, it is impossible to prove its long-term efficacy due to the lack of direct evidence.

The dental transverse expansion was measured at the maxillary canines, first premolar, second premolar, first molar in 10 articles, and the mean ICW was 3.36 mm (2.03–4.69 mm), the mean IP1W was 4.23 mm (3.48–4.97 mm), the mean IP2W was 4.01 mm (3.17–4.85 mm), and the mean IMW was 4.79 mm (3.35–6.23 mm). These results indicated that the dental transverse expansion is a "V" shaped expansion, which is anteriorly narrow and posteriorly wide in the horizontal plane. In particular, Kapetanović et al. reported that the mean IMW increase was 6.55 mm (5.50–7.59 mm), which was greater than the results reported in this study. This suggests that a certain degree of relapse occurs in the expansion over time.

Except for two outcomes (IP1M and IP2M), the other outcomes (skeletal and dental expansions) showed that the effect of the expansion was worse for the long follow-up interval, which indicated that expansion would be accompanied by a degree of relapse. Tang et al. reported that the expanded skeletal width was generally stable after the MARPE procedure, but a certain amount of relapse occurred over time [19], which is consistent with this study. Lim et al*.* also assessed the differences in dental, alveolar, and skeletal measurements taken before, immediately after, and 1 year after MARPE. The changes of intercusp, interapex, alveolar, nasal floor, and nasal cavity widths; inclination of the first molar and its alveolus; and thickness and height of the alveolar bone were measured. The MARPE procedure showed stable outcomes 1 year after expansion and produced significant increases in 1 year after expansion, despite the relapse of some measurements from immediately after expansion to 1 year after expansion [34]. The two outcomes (IP1M and IP2M) did not show a relapse trend, which may be due to the small number of articles included.

The mean basal bone composition accounted for 48.85% of the total expansion, the mean alveolar bone accounted for 7.52% and the mean dental expansion accounted for 43.63% in at least two months after expansion. The basal bone composition, mean alveolar bone and mean dental expansion accounted for 47.55, 10.02, and 42.43% in at least four months after expansion, indicating that the changes in skeletal width are generally stable for two months and longer after MARPE treatment. Kapetanović et al. reported that the mean skeletal component of MARPE was 35.6% [20], which is worse than that found in this study. The reason for this discrepancy may be that the dental relapse degree is greater than that of maxillary.

The definition of the buccal inclination of the teeth varied slightly. Three studies measured the angle of the tooth axis to the hard palate or nasal floor [15, 30, 34]. Alsayegh et al*.* measured the angle of intersection of the lines drawn tangent to the mesio-facial and mesio-palatal cusp tips of the maxillary first molars [31]. McMullen et al. measured the angle change of the long axis of the molars before and after treatment by constructing a 3D coordinate system and superimposing the anterior cranial bases [32]. Calil et al. calculated the angle between the line passing through the long axis of the tooth and the vertical line parallel to the midsagittal plane [33]. The maxillary first molars were the most common, with a mean value ranging from 0.6° to 4.9°, and two were statistically significant [15, 30]. Compared with a previously reported study of the buccal inclination of 2.07° to 8.01° (all statistically significant) [20], there is a difference, which may be mainly due to a certain level of relapse of the dental width.

Alveolar and periodontal side effects were reported in four articles [15, 30, 33, 34]. The buccal inclination of the alveolar bone, reported in three articles, was commonly calculated by measuring the angle between the palatal alveolar bone and nasal floor or palatal floor, with a mean range from 0.4° to 2.26°, and all were statistically significant [15, 30, 34]. A mean decrease of the alveolar crest level at M1 was reported in three articles, ranging from 0.11 to 0.8 mm [15, 30, 34], and all but one were statistically significant [34]. Additionally, two articles reported a decrease of the alveolar bone thickness on the buccal side and (or) the palatal side, ranging 0.13 to 0.33 mm, which were all not statistically significant [33, 34]. This finding suggests that MARPE mainly causes buccal inclination of the alveolar bone, but has little effect on the alveolar bone thickness. However, it may make a difference in the alveolar crest level. The risk of periodontal side effects will increase in patients with a compromised periodontal situation, which suggests that orthodontists should beware of that.

The changes of other craniofacial bones were reported in five studies, including the sphenoid bone (usually refers to lateral pterygoid plate), temporal bones and zygomatic bones [19, 27, 30, 32, 35]. However, the measurement methods varied across studies. The zygomatic bone was the most reported as having no statistical significance [27, 32, 35]. McMullen et al. reported the change of the orbital point, which was not statistically significant. The changes in the other craniofacial bones were all statistically significant. This suggests that with the expansion of the nasomaxillary complex, the connective tissues of the sutures of the skulls will undergo a certain degree of bone remodeling. Whether this is clinically significant needs further research.

Changes of the upper airway were reported in two articles [28, 29]. Yi et al. reported that the nasopharyngeal volume was significantly increased by 8.48% after MARPE treatment compared with that before the treatment (*P* < *0.05*), but there was no statistically significant change in the oropharyngeal, palatopharyngeal, glossopharyngeal and airway total volume (all *P* > *0.05*) [29]. According to Li Q et al., the volume of the nasal cavity and nasopharynx increased significantly *(P* < *0.05)*, and the enlarged nasopharyngeal volume was correlated with the increased nasal width at the PNS plane (*P* < *0.05*), but there was no correlation between the expanded volume and maxillary width [28]. Based on these studies, we can draw a conclusion that the MARPE treatment can improve the upper airway ventilation.

An et al. reported changes in facial soft tissues at 2.91 ± 0.59 months after expansion[27], and only the changes in the alar width and the nose length were statistically significant in the range of (1.18 ± 1.52) % and (0.98 ± 2.32) %, respectively. Ramieri et al. reported that the magnitude of facial changes was limited but clinically significant at 1 year after SARPE, with a cutaneous changes in the paranasal regions and cheeks (range 1–3 mm), and with a significant enlargement of the nasal base [39]. In general, there was basically no significant soft tissue change after MARPE, and further research on the long-term facial changes is needed.

### Limitations and future prospects

The limitations of the present systematic study using meta-analysis are mainly in the following three aspects. First, seven studies were at a serious risk of bias, and the overall quality of the evidence was assessed as very low. As a result of the observational studies included and the lack of high-quality studies, it is not possible to draw strong conclusions. In addition, the design of the device, the orientation of the jackscrew, and the expansion protocol were similar but different, which may have a significant impact. Furthermore, there were great differences in methodology, measurement method, physiological age of subjects and other factors that may affect the results. High-quality studies are necessary to obtain a higher quality of evidence on the efficacy of the MARPE treatment. Most studies published before the search date typically have follow-up dates of 3–6 months, we are unable to obtain longer term data after MARPE. We hope that more research will be devoted to studying the long-term efficacy of MARPE in the future.

Most studies were designed as observational studies that cannot adjust for known confounding factors, resulting in relatively low quality of evidence. Further and longer-term research is needed to improve the quality of evidence. The timing of palatal expansion is best determined by the suture fusion. However, most studies typically group based on age. In addition, the length of the miniscrew and the depth of implantation, expansion protocol, indicators for the success of the expansion, and measurements all vary. We hope that more research will be conducted to explore and obtain a more efficient and detailed MARPE, in order to obtain higher quality research.

## Conclusions

The systematic review and meta-analysis of the long-term efficacy and stability demonstrated that:

1. MARPE has proven to be a successful treatment for transverse maxillary deficiency (mean success rate: 93.87%).
2. Skeletal transverse expansion of the nasomaxillary complex is embodied in "A" type in four aspects: maxillary basal bone increased mean 2.34 mm (95%CI: 1.71–2.97 mm), maxillary alveolar bone increased mean 2.70 mm (95%CI: 2.11–3.30 mm), nasal floor width increased mean 2.18 mm (95%CI: 1.71–2.66 mm) and nasal lateral width increased mean 1.96 mm (95%CI: 1.43–2.49 mm).
3. Dental transverse expansion is a "V" shaped expansion: the ICW increased mean was 3.36 mm (95%CI: 2.03–4.69 mm), the IP1W increased mean was 4.23 mm (95%CI: 3.48–4.97 mm), the IP2W increased mean was 4.01 mm (95%CI: 3.17–4.85 mm), the IMW increased mean was 4.79 mm (95%CI: 3.35–6.23 mm).
4. The basal bone composition accounted for 48.85% of the total expansion, the mean alveolar bone accounted for 7.52% and the mean dental expansion accounted for 43.63%.
5. Changes in skeletal and dental width were generally stable after MARPE treatment, although a certain degree of relapse occurs over time for both skeletal and dental expansion with MARPE.
6. MARPE may cause dental, alveolar and periodontal side effects, and have an impact on other craniofacial bones, upper airway, and facial soft tissue.

### Supplementary Information

**Additional file 1:** **Supplementary Table 1.** Search strategy used for electronic database search.

**Additional file 2:** **Supplementary Table 2.** List of excluded studies.

**Additional file 3:** **Supplementary Table 3.** Results of individual studies for dental side effects at M1 by MARPE.

**Additional file 4: Supplementary Table 4a.** Results of individual studies for periodontal side effects (buccal inclination of alveolar bone at M1) by MARPE. **Table 4b.** Results of individual studies for periodontal side effects (buccal alveolar crest level at M1) by MARPE.

**Additional file 5: Supplementary Table 5.** Results of individual studies for craniofacial bone change by MARPE.

**Additional file 6: Supplementary Table 6.** Risk of bias assessment across studies according to the GRADE methodology.

## Data Availability

All data generated or analyzed during this study are included in this published article.

## Abbreviations

MARPE: Miniscrew-assisted Rapid Palatal Expansion
MPS: Mid-palatal suture
RME: Rapid maxillary expansion
SARME: Surgically-assisted rapid maxillary expansion
PRISMA: Preferred Reporting Items for Systematic Reviews and Meta-Analyses
CNKI: Chinese National Knowledge Infrastructure
ROB: Revised Cochrane Risk of Bias Tool for Randomized Trials
ROBINS-I: Risk Of Bias in Non-randomized Studies of Interventions
MDs: Mean differences
CIs: Corresponding 95% confidence intervals
GRADE: Grading of Recommendations Assessment, Development and Evaluation
CVS: Cervical spine staging
CBCT: Cone beam computed tomography
M1: First molar
ICW: Intercanine width
IP1W: Inter-first premolar width
IP2W: Inter-second premolar width
IMW: Intermolar width
PNS: Posterior nasal spine
C: Canines
P1: First premolar
P2: Second premolar
