# Supplementary material for: Long-term efficacy and stability of miniscrew-assisted rapid palatal expansion in mid to late adolescents and adults: a systematic review and meta-analysis
Source: BMC Oral Health. 2023 Nov 3;23:829. doi: 10.1186/s12903-023-03574-y (PMC10623697; doi:10.1186/s12903-023-03574-y)
Supplement: Supplementary file 1 — Additional file 1: Supplementary Table 1. Search strategy used for electronic database search. [file 12903_2023_3574_MOESM1_ESM.docx]

**Supplementary Table 1.** Search strategy used for electronic database search.

| **Database** | **Search terms** |
| --- | --- |
| MEDLINE  (via PubMed) | (((bone screws[MeSH Terms])OR ((screw*[Title/Abstract])AND (bone[Title/Abstract]))OR (bone-anchored[Title/Abstract])OR (bone-borne[Title/Abstract])OR (implant anchorage*[Title/Abstract])OR (implant-supported[Title/Abstract])OR (miniimplant*[Title/Abstract])OR (micro implant*[Title/Abstract])OR (micro screw*[Title/Abstract])OR (mini implant*[Title/Abstract])OR (mini screw*[Title/Abstract])OR (mini-implant*[Title/Abstract])OR (miniscrew*[Title/Abstract])OR (mini-screw*[Title/Abstract])OR (orthodontic anchorage*[Title/Abstract])OR (orthodontic anchorage procedure*[Title/Abstract])OR (orthodontic anchorage technique*[Title/Abstract])OR (orthodontic anchoring procedure*[Title/Abstract])OR (skeletal anchorage*[Title/Abstract])OR (anchorage screw*[Title/Abstract])OR (temporary anchorage device*[Title/Abstract])OR ((anchorage procedure*[Title/Abstract])AND (orthodontic[Title/Abstract]))OR ((anchorage technique*[Title/Abstract])AND (orthodontic[Title/Abstract]))OR ((procedure*[Title/Abstract])AND (orthodontic anchorage*[Title/Abstract]))OR ((technique*[Title/Abstract])AND (orthodontic anchorage*[Title/Abstract]))OR (TAD[Title/Abstract])OR (TADs[Title/Abstract]))AND ((palatal expansion technique*[MeSH Terms])OR (palatal expansion technic*[Title/Abstract])OR (palatal expander*[Title/Abstract])OR (palatal expansion*[Title/Abstract])OR (maxilla expansion*[Title/Abstract])OR (maxillary expansion*[Title/Abstract])OR (maxillary suture expansion*[Title/Abstract])OR ((expansion*[Title/Abstract])AND (maxillary[Title/Abstract]))OR ((expansion technic*[Title/Abstract])AND (palatal[Title/Abstract]))OR ((expansion technique*[Title/Abstract])AND (palatal[Title/Abstract]))OR ((technic*[Title/Abstract])AND (palatal expansion*[Title/Abstract]))OR ((technique*[Title/Abstract])AND (palatal expansion*[Title/Abstract]))))OR (((MARPE[Title/Abstract])OR (MARME[Title/Abstract]))) |
| EMBASE | 'marme':ti, ab, kw OR 'marpe':ti, ab, kw OR (('palatal expansion'/exp OR ('expansion technique*':ti, ab, kw AND palatal:ti, ab, kw)OR 'palatal expansion technique*':ti, ab, kw OR (technique*:ti, ab, kw AND 'palatal expansion*':ti, ab, kw)OR 'palatal expansion technic*':ti, ab, kw OR ('expansion technic*':ti, ab, kw AND palatal:ti, ab, kw)OR 'palatal expansion technic*':ti, ab, kw OR (technic*:ti, ab, kw AND 'palatal expansion*':ti, ab, kw)OR 'maxillary expansion*':ti, ab, kw OR (expansion*:ti, ab, kw AND maxillary:ti, ab, kw)OR 'palatal expansion technique*':ti, ab, kw OR 'palatal expansion*':ti, ab, kw OR 'palatal expander*':ti, ab, kw OR 'maxilla expansion*':ti, ab, kw OR 'maxillary suture expansion*':ti, ab, kw AND ('bone screw'/exp OR (screw*:ti, ab, kw AND bone:ti, ab, kw)OR miniscrew*:ti, ab, kw OR miniimplant*:ti, ab, kw OR 'micro screw*':ti, ab, kw OR 'skeletal anchorage*':ti, ab, kw OR tad:ti, ab, kw OR tads:ti, ab, kw OR 'temporary anchorage device*': ti, ab, kw OR 'anchorage screw*':ti, ab, kw OR 'micro implant*':ti, ab, kw OR 'mini implant*':ti, ab, kw OR 'mini screw*':ti, ab, kw OR 'implant supported':ti, ab, kw OR 'implant anchorage*':ti, ab, kw OR 'orthodontic anchorage*':ti, ab, kw OR 'bone borne':ti, ab, kw OR 'bone anchored':ti, ab, kw OR 'orthodontic anchorage procedure*':ti, ab, kw OR 'orthodontic anchoring procedure*':ti, ab, kw OR 'orthodontic anchorage technique*':ti, ab, kw OR (technique*:ti, ab, kw AND 'orthodontic anchorage':ti, ab, kw)OR ('anchorage technique*':ti, ab, kw AND orthodontic:ti, ab, kw)OR (procedure*:ti, ab, kw AND 'orthodontic anchorage':ti, ab, kw)OR ('anchorage procedure*':ti, ab, kw AND orthodontic:ti, ab, kw)) |
| Cochrane Library | #1 MeSH descriptor: [Palatal Expansion Technique] explode all trees  #2 Palatal Expansion* Technique*  #3 Expansion* Technic* Palatal  #4 Expansion* Maxillary  #5 Maxilla* Expansion*  #6 Maxillary suture expansion*  #7 palatal expander*  #8 palatal expansion*  #9 #1 OR #2 OR #3 OR #4 OR #5 OR #6 OR #7 OR #8  #10 anchorage procedure* orthodontic  #11 anchorage screw*  #12 anchorage technique* orthodontic  #13 bone screw*  #14 bone-anchored  #15 bone-borne  #16 miniimplant*  #17 implant anchorage*  #18 implant-supported  #19 micro implant*  #20 micro screw*  #21 mini implant*  #22 mini screw*  #23 mini-implant*  #24 miniscrew*  #25 mini-screw*  #26 orthodontic anchorage*  #27 orthodontic anchorage procedure*  #28 orthodontic anchorage technique*  #29 orthodontic anchoring procedure*  #30 screw* bone  #31 skeletal anchorage*  #32 tad  #33 tads  #34 temporary anchorage device*  #35 #10 OR #11 OR # 12 OR #13 OR #14 OR #15 OR #16 OR #17 OR #18 OR #19 OR #20 OR #21 OR #22 OR #23 OR #24 OR #25 OR #26 OR #27 OR #28 OR #29 OR #30 OR #31 OR #32 OR #33 OR #34  #36 #9 AND #35  #37 MARPE  #38 MARME  #39 #36 OR #37 OR #38 |
| Web of Science | #1 ((((((((((((((((((((((((((TS=(bone screws))OR TS=(screw* bone))OR TS=(bone-anchored))OR TS=(bone-borne))OR TS=(implant anchorage*))OR TS=(implant-supported))OR TS=(miniimplant*))OR TS=(micro implant*))OR TS=(micro screw*))OR TS=(mini implant*))OR TS=(mini screw*))OR TS=(mini-implant*))OR TS=(miniscrew*))OR TS=(mini-screw*))OR TS=(orthodontic anchorage*))OR TS=("orthodontic anchorage procedure*"))OR TS=("orthodontic anchorage technique*"))OR TS=("orthodontic anchoring procedure*"))OR TS=(skeletal anchorage*))OR TS=(anchorage screw*))OR TS=("temporary anchorage device*"))OR TS=("anchorage procedure*" orthodontic))OR TS=("anchorage technique*" orthodontic))OR TS=(procedure* "orthodontic anchorage*"))OR TS=(technique* "orthodontic anchorage*"))OR TS=(TAD))OR TS=(TADs)  #2 ((((((((((TS=("palatal expansion technique*"))OR TS=(palatal expansion technic*))OR TS=(palatal expander*))OR TS=(palatal expansion*))OR TS=(maxilla expansion*))OR TS=(maxillary expansion*))OR TS=("maxillary suture expansion*"))OR TS=("expansion technic*" palatal))OR TS=("expansion technique*" palatal))OR TS=(technic* "palatal expansion*"))OR TS=(technique* "palatal expansion*")  #3 (TS=(MARPE))OR TS=(MARME)  #4 #2 AND #1  #5 #4 OR #3 |
| Scopus | ((TITLE-ABS-KEY (marpe ) OR TITLE-ABS-KEY (marme ))) OR (((TITLE-ABS-KEY ("bone screws" )) OR (TITLE-ABS-KEY (screw* AND bone )) OR (TITLE-ABS-KEY (bone-anchored )) OR (TITLE-ABS-KEY (bone-borne )) OR (TITLE-ABS-KEY (implant AND anchorage* )) OR (TITLE-ABS-KEY (implant-supported )) OR (TITLE-ABS-KEY (miniimplant* )) OR (TITLE-ABS-KEY (micro AND implant* )) OR (TITLE-ABS-KEY (micro AND screw* )) OR (TITLE-ABS-KEY (mini AND implant* )) OR (TITLE-ABS-KEY (mini AND screw* )) OR (TITLE-ABS-KEY (mini-implant* )) OR (TITLE-ABS-KEY (miniscrew* )) OR (TITLE-ABS-KEY (mini-screw* )) OR (TITLE-ABS-KEY (orthodontic AND anchorage* )) OR (TITLE-ABS-KEY ("orthodontic anchorage procedure*" )) OR (TITLE-ABS-KEY ("orthodontic anchorage technique*" )) OR (TITLE-ABS-KEY ("orthodontic anchoring procedure*" )) OR (TITLE-ABS-KEY ("orthodontic anchoring procedure*" )) OR (TITLE-ABS-KEY (skeletal AND anchorage* )) OR (TITLE-ABS-KEY (anchorage AND screw* )) OR (TITLE-ABS-KEY (temporary AND anchorage AND device* )) OR (TITLE-ABS-KEY ("anchorage procedure*" orthodontic )) OR (TITLE-ABS-KEY ("anchorage technique*" orthodontic )) OR (TITLE-ABS-KEY (procedure* "orthodontic anchorage*" )) OR (TITLE-ABS-KEY (technique* "orthodontic anchorage*" )) OR (TITLE-ABS-KEY (tad )) OR (TITLE-ABS-KEY (tads ))) AND ((TITLE-ABS-KEY ("palatal expansion technique*" )) OR (TITLE-ABS-KEY (palatal AND expansion AND technic* )) OR (TITLE-ABS-KEY (palatal AND expander* )) OR (TITLE-ABS-KEY (palatal AND expansion* )) OR (TITLE-ABS-KEY (maxilla AND expansion* )) OR (TITLE-ABS-KEY (maxillary AND suture AND expansion* )) OR (TITLE-ABS-KEY (expansion* AND maxillary )) OR (TITLE-ABS-KEY ("expansion technic*" palatal )) OR (TITLE-ABS-KEY ("expansion technique*" palatal )) OR (TITLE-ABS-KEY (technic* "palatal expansion*" )) OR (TITLE-ABS-KEY (technique* "palatal expansion*" )))) |
| Chinese National Knowledge Infrastructure (CNKI) | (TI= 'miniscrew (in Chinese)' OR TKA= 'TAD'+'TADs' AND TI= 'expansion (in Chinese)' AND TI= 'maxillary(in Chinese)' ) OR TKA= 'MARPE'+'MARPE'+'MSE' |
| Wanfang | (Title or Keywords: ('miniscrew (in Chinese)' or 'TAD' or 'TADs' and Title or Keywords:('expansion (in Chinese)' and 'maxillary(in Chinese)'))or Title or Keywords:('MARPE' or 'MARPE' or 'MSE') |
