# Supplementary material for: Long-term efficacy and stability of miniscrew-assisted rapid palatal expansion in mid to late adolescents and adults: a systematic review and meta-analysis
Source: BMC Oral Health. 2023 Nov 3;23:829. doi: 10.1186/s12903-023-03574-y (PMC10623697; doi:10.1186/s12903-023-03574-y)
Supplement: Supplementary file 2 — Additional file 2: Supplementary Table 2. List of excluded studies. [file 12903_2023_3574_MOESM2_ESM.docx]

**Supplementary Table 2.** List of excluded studies.

| **Study** | **Exclusion** |
| --- | --- |
| Long-term effects on alveolar bone with bone-anchored and tooth-anchored rapid palatal expansion | Patient population (< 13.5 years of age). |
| Long-term effects of conventional and miniscrew-assisted rapid palatal expansion on root resorption | Patient population (< 13.5 years of age). |
| Skeletal and alveolar changes in conventional rapid palatal expansion (RPE) and miniscrew-assisted RPE (MARPE): a prospective randomized clinical trial using low-dose CBCT | Patient population (< 13.5 years of age). |
| Comparison of changes in skeletal, dentoalveolar, periodontal, and nasal structures after tooth-borne or bone-borne rapid maxillary expansion: A parallel cohort study | Patient population (< 13.5 years of age). |
| Computational fluid dynamics analysis of nasal airway changes after treatment with C-expander | No relevant results. |
| Midfacial soft tissue changes after maxillary expansion using micro-implant-supported maxillary skeletal expanders in young adults: A retrospective study | No relevant results. |
| Long-term effects of mini-screw–assisted rapid palatal expansion on airway: A three-dimensional cone-beam computed tomography study | Patient population (< 13.5 years of age). |
| Long-term assessment of conventional and mini-screw-assisted rapid palatal expansion on the nasal cavity | Patient population (< 13.5 years of age). |
| Bone-anchored versus tooth-anchored expansion appliances: Long-term effects on the condyle–fossa relationship | Patient population (< 13.5 years of age). |
| The effect of skeletal and dental expansion according to the presence or absence of miniscrews in the treatment of palatal expansion | Patient population (< 13.5 years of age). |
| A postero-anterior cephalometric evaluation of different rapid maxillary expansion appliances | Patient population (< 13.5 years of age). |
| Orthopedic outcomes of hybrid and conventional Hyrax expanders: secondary data analysis from a randomized clinical trial | Patient population (< 13.5 years of age). |
| Dentoskeletal changes due to rapid maxillary expansion in growing patients with tooth-borne and tooth-bone-borne expanders: a randomized clinical trial | Patient population (< 13.5 years of age). |
| Skeletal and dentoalveolar effects using tooth-borne and tooth-bone-borne RME appliances: a randomized controlled trial with 1-year follow-up | No relevant results. |
| Differential assessment of skeletal, alveolar, and dental components induced by microimplant-supported midfacial skeletal expander (MSE), utilizing novel angular measurements from the fulcrum | Patient population (< 13.5 years of age). |
| Molar inclination and surrounding alveolar bone change relative to the design of bone-borne maxillary expanders: A CBCT study | No relevant results. |
| Evaluation of the changes of orbital cavity volume and shape after tooth-borne and bone-borne rapid maxillary expansion (RME) | Patient population (< 13.5 years of age). |
| Transverse dentoalveolar response of mandibular arch after rapid maxillary expansion (RME) with tooth-borne and bone-borne appliances | Patient population (< 13.5 years of age). |
| Transverse, vertical, and anterior-posterior changes between tooth-anchored versus Dresden bone-anchored rapid maxillary expansion 6 months post-expansion: A CBCT randomized controlled clinical trial | Patient population (< 13.5 years of age). |
| Miniscrew assisted rapid palatal expander- an alternative to conventional expanders | No relevant results. |
| Long term skeletal and dental changes between tooth-anchored versus Dresden bone-anchored rapid maxillary expansion using CBCT images in adolescents: Randomized clinical trial | Patient population (< 13.5 years of age). |
| Rapid palatal expander: Maxillary and mandibular measurements using manual and digital methods | No relevant results. |
| Three-dimensional changes of the zygomaticomaxillary complex after mini-implant assisted rapid maxillary expansion | Patient population (< 13.5 years of age). |
| Comparison of traditional RPE with two types of micro-implant assisted RPE: CBCT study | No relevant results. |
| Asymmetric nasomaxillary expansion induced by tooth-bone-borne expander producing differential craniofacial changes | Patient population (< 13.5 years of age). |
| Retrospective CBCT analysis of airway volume changes after bone-borne vs tooth-borne rapid maxillary expansion | Patient population (< 13.5 years of age). |
| Evaluation of miniscrew-supported rapid maxillary expansion in adolescents: A prospective randomized clinical trial | No relevant results. |
| Skeletal and dentoalveolar changes after miniscrew-assisted rapid palatal expansion in young adults: A cone-beam computed tomography study | Patient population (< 13.5 years of age). |
| Comparison of the treatment effects of different rapid maxillary expansion devices on the maxilla and the mandible. Part 1: Evaluation of dentoalveolar changes | Patient population (< 13.5 years of age). |
| Dentoskeletal effects of a temporary skeletal anchorage device-supported rapid maxillary expansion appliance (TSADRME): A pilot study | No relevant results. |
| The maxillary expansion with different devices | Patient population (unclear age). |
| Periodontal, dentoalveolar, and skeletal effects of tooth-borne and tooth-bone-borne expansion appliances | Patient population (< 13.5 years of age). |
| Short-term skeletal and dental changes following bone-borne versus tooth-borne surgically assisted rapid maxillary expansion: a randomized clinical trial study | No relevant results. |
| Transverse, vertical, and anteroposterior changes from bone-anchored maxillary expansion vs traditional rapid maxillary expansion: a randomized clinical trial | Patient population (< 13.5 years of age). |
| Minimally invasive rapid palatal expansion with an implant-supported hyrax screw | No relevant results. |
| Transverse maxillary distraction in patients with periodontal pathology or insufficient tooth anchorage using custom-made devices | No relevant results. |
| Evaluation on expansion efficiency of microimplant-assisted rapid palatal expansion in treatment of maxillary transverse deficiency in adolescents with CBCT | No relevant results. |
| CBCT study on the effect of MSE on the maxillary first molar and alveolar bone (in Chinese) | Patient population (< 13.5 years of age). |
| The Effect of Maxillary Skeletal Expander on the Treatment of Maxillary Transverse Deficiency | Patient population (< 13.5 years of age). |
| Clinical analysis of microimplant assisted maxillary rapid expansion combined with fixation | No relevant results. |
| Clinical Analysis of Maxillary Rapid Expansion Using Implant Assisted Nail | No relevant results. |
| Long-term effects on alveolar bone with bone-anchored and tooth-anchored rapid palatal expansion | Patient population (< 13.5 years of age). |
| Long-term effects of conventional and miniscrew-assisted rapid palatal expansion on root resorption | Patient population (< 13.5 years of age). |
| Skeletal and alveolar changes in conventional rapid palatal expansion (RPE) and miniscrew-assisted RPE (MARPE): a prospective randomized clinical trial using low-dose CBCT | Patient population (< 13.5 years of age). |
| Comparison of changes in skeletal, dentoalveolar, periodontal, and nasal structures after tooth-borne or bone-borne rapid maxillary expansion: A parallel cohort study | Patient population (< 13.5 years of age). |
| Computational fluid dynamics analysis of nasal airway changes after treatment with C-expander | No relevant results. |
| Midfacial soft tissue changes after maxillary expansion using micro-implant-supported maxillary skeletal expanders in young adults: A retrospective study | No relevant results. |
| Long-term effects of mini-screw–assisted rapid palatal expansion on airway: A three-dimensional cone-beam computed tomography study | Patient population (< 13.5 years of age). |
| Long-term assessment of conventional and mini-screw-assisted rapid palatal expansion on the nasal cavity | Patient population (< 13.5 years of age). |
| Bone-anchored versus tooth-anchored expansion appliances: Long-term effects on the condyle–fossa relationship | Patient population (< 13.5 years of age). |
| The effect of skeletal and dental expansion according to the presence or absence of miniscrews in the treatment of palatal expansion | Patient population (< 13.5 years of age). |
| A postero-anterior cephalometric evaluation of different rapid maxillary expansion appliances | Patient population (< 13.5 years of age). |
| Orthopedic outcomes of hybrid and conventional Hyrax expanders: secondary data analysis from a randomized clinical trial | Patient population (< 13.5 years of age). |
| Dentoskeletal changes due to rapid maxillary expansion in growing patients with tooth-borne and tooth-bone-borne expanders: a randomized clinical trial | Patient population (< 13.5 years of age). |
| Skeletal and dentoalveolar effects using tooth-borne and tooth-bone-borne RME appliances: a randomized controlled trial with 1-year follow-up | No relevant results. |
| Differential assessment of skeletal, alveolar, and dental components induced by microimplant-supported midfacial skeletal expander (MSE), utilizing novel angular measurements from the fulcrum | Patient population (< 13.5 years of age). |
| Molar inclination and surrounding alveolar bone change relative to the design of bone-borne maxillary expanders: A CBCT study | No relevant results. |
| Evaluation of the changes of orbital cavity volume and shape after tooth-borne and bone-borne rapid maxillary expansion (RME) | Patient population (< 13.5 years of age). |
| Transverse dentoalveolar response of mandibular arch after rapid maxillary expansion (RME) with tooth-borne and bone-borne appliances | Patient population (< 13.5 years of age). |
| Transverse, vertical, and anterior-posterior changes between tooth-anchored versus Dresden bone-anchored rapid maxillary expansion 6 months post-expansion: A CBCT randomized controlled clinical trial | Patient population (< 13.5 years of age). |
| Miniscrew assisted rapid palatal expander- an alternative to conventional expanders | No relevant results. |
| Long term skeletal and dental changes between tooth-anchored versus Dresden bone-anchored rapid maxillary expansion using CBCT images in adolescents: Randomized clinical trial | Patient population (< 13.5 years of age). |
| Rapid palatal expander: Maxillary and mandibular measurements using manual and digital methods | No relevant results. |
| Three-dimensional changes of the zygomaticomaxillary complex after mini-implant assisted rapid maxillary expansion | Patient population (< 13.5 years of age). |
| Comparison of traditional RPE with two types of micro-implant assisted RPE: CBCT study | No relevant results. |
| Asymmetric nasomaxillary expansion induced by tooth-bone-borne expander producing differential craniofacial changes | Patient population (< 13.5 years of age). |
| Retrospective CBCT analysis of airway volume changes after bone-borne vs tooth-borne rapid maxillary expansion | Patient population (< 13.5 years of age). |
| Evaluation of miniscrew-supported rapid maxillary expansion in adolescents: A prospective randomized clinical trial | No relevant results. |
| Skeletal and dentoalveolar changes after miniscrew-assisted rapid palatal expansion in young adults: A cone-beam computed tomography study | Patient population (< 13.5 years of age). |
| Comparison of the treatment effects of different rapid maxillary expansion devices on the maxilla and the mandible. Part 1: Evaluation of dentoalveolar changes | Patient population (< 13.5 years of age). |
| Dentoskeletal effects of a temporary skeletal anchorage device-supported rapid maxillary expansion appliance (TSADRME): A pilot study | No relevant results. |
| The maxillary expansion with different devices | Patient population (unclear age). |
| Periodontal, dentoalveolar, and skeletal effects of tooth-borne and tooth-bone-borne expansion appliances | Patient population (< 13.5 years of age). |
| Short-term skeletal and dental changes following bone-borne versus tooth-borne surgically assisted rapid maxillary expansion: a randomized clinical trial study | No relevant results. |
| Transverse, vertical, and anteroposterior changes from bone-anchored maxillary expansion vs traditional rapid maxillary expansion: a randomized clinical trial | Patient population (< 13.5 years of age). |
| Minimally invasive rapid palatal expansion with an implant-supported hyrax screw | No relevant results. |
| Transverse maxillary distraction in patients with periodontal pathology or insufficient tooth anchorage using custom-made devices | No relevant results. |
| Evaluation on expansion efficiency of microimplant-assisted rapid palatal expansion in treatment of maxillary transverse deficiency in adolescents with CBCT | No relevant results. |
| CBCT study on the effect of MSE on the maxillary first molar and alveolar bone | Patient population (< 13.5 years of age). |
| The Effect of Maxillary Skeletal Expander on the Treatment of Maxillary Transverse Deficiency | Patient population (< 13.5 years of age). |
| Clinical analysis of microimplant assisted maxillary rapid expansion combined with fixation | No relevant results. |
| Clinical Analysis of Maxillary Rapid Expansion Using Implant Assisted Nail | No relevant results. |
| [Nasomaxillary Expansion by Endoscopically-Assisted Surgical Expansion (EASE): An airway centric approach] | No relevant results. |
| [Persistent pediatric obstructive sleep apnea treated with skeletally anchored transpalatal distraction] | No relevant results. |
| Aligner hybrid orthodontic approach to treat severe transverse divergence in an adolescent girl: A case report | No relevant results. |
| An evaluation of three-dimensional facial changes after surgically assisted rapid maxillary expansion (SARME): an observational study | No relevant results. |
| Assessment of the bone thickness of the palate on cone-beam computed tomography for placement of miniscrew-assisted rapid palatal expansion appliances | No relevant results. |
| Cephalometric and model evaluations after molar distalization using modified C-palatal plates in patients with severe arch length discrepancy | No relevant results. |
| Clinical and Radiographic Outcomes of a Novel Transalveolar Sinus Floor Elevation Technique | No relevant results. |
| Clinical application of maxillary tissue bone-borne expander and biocreative reverse curve system in the orthodontic retreatment of severe anterior open bite with transverse discrepancy: A case report | No relevant results. |
| Comparative efficacy of the bone-anchored maxillary protraction protocols for orthopaedic treatment in skeletal Class III malocclusion: A Bayesian network meta-analysis | No relevant results. |
| Comparison of facemask therapy effects using skeletal and tooth-borne anchorage | No relevant results. |
| Comparison of the short-term effects of tooth-bone-borne and tooth-borne rapid maxillary expansion in older adolescents | No relevant results. |
| Condylar morphology and position changes after miniscrew-assisted rapid palatal expansion in skeletal Class III malocclusion adult patients with mandibular deviation and unilateral posterior crossbite | No relevant results. |
| Correction of a true full-cusp unilateral posterior crossbite using mini-screw assisted biomechanics in an adult hyperdivergent patient | No relevant results. |
| Dentoskeletal changes due to rapid maxillary expansion in growing patients with tooth-borne and tooth-bone-borne expanders: A randomized clinical trial | Patient population (< 13.5 years of age). |
| Do Gender and Age Influence Nasal Soft Tissue Widening After Bone-Borne Transpalatal Distraction? | No relevant results. |
| Does MARPE therapy have effects on intracranial pressure? a clinical study | No relevant results. |
| Early Class III Treatment Using a Hybrid Rapid Palatal Expander and Facemask in a Patient with Partially Edentulous Maxilla Post MNTI Removal: A Case Report | No relevant results. |
| Effect and stability of miniscrew-assisted rapid palatal expansion: A systematic review and meta-analysis | systematic review and meta-analysis |
| Effectiveness of miniscrew assisted rapid palatal expansion using cone beam computed tomography: A systematic review and meta-analysis | systematic review and meta-analysis |
| Effects of miniscrew location on biomechanical performances of bone-borne rapid palatal expander to midpalatal suture: A finite element study | finite element study |
| Efficacy of Miniscrew-Assisted Rapid Palatal Expansion (MARPE) in late adolescents and adults with the Dutch Maxillary Expansion Device: a prospective clinical cohort study | No relevant results. |
| Efficacy of the Miniscrew-Assisted Rapid Palatal Expansion: An Original Research | No relevant results. |
| Evaluation of bone depth, cortical bone, and mucosa thickness of palatal posterior supra-alveolar insertion site for miniscrew placement | No relevant results. |
| Evaluation of RANK, RANKL and OPG Levels in Gingival Crevicular Fluid After Surgically Assisted Rapid Maxillary Expansion | No relevant results. |
| Influence of the palatal plane cant and skeletal patterns in the hard palate thickness? | No relevant results. |
| Interest of miniscrew-assisted rapid palatal expansion on the upper airway in growing patients: A systematic review | systematic review |
| Long-term assessment of conventional and mini-screw-assisted rapid palatal expansion on the nasal cavity | Patient population (< 13.5 years of age). |
| Long-term skeletal and dentoalveolar effects of hybrid rapid maxillary expansion and facemask treatment in growing skeletal Class III patients: a retrospective follow-up study | No relevant results. |
| Management of Class III Malocclusion and Maxillary Transverse Deficiency with Microimplant-Assisted Rapid Palatal Expansion (MARPE): A Case Report | No relevant results. |
| Midpalatal suture bone repair after miniscrew-assisted rapid palatal expansion in adults | No relevant results. |
| Patient-reported outcomes of slow vs rapid miniscrew-supported maxillary expansion in adolescents: secondary outcomes of a randomized clinical trial | No relevant results. |
| Polygraphic evaluation of the effects of different rapid maxillary expansion appliances on sleep quality: A randomized clinical trial | Patient population (< 13.5 years of age). |
| Radiographic and histological assessment of root resorption associated with conventional and mini-screw assisted rapid palatal expansion: a systematic review | systematic review |
| Rapid Maxillary Expansion on the Adolescent Patient: Systematic Review and Case Report | systematic review |
| Skeletal and dentoalveolar effects of slow vs rapid activation protocols of miniscrew-supported maxillary expanders in adolescents: A randomized clinical trial | Patient population (< 13.5 years of age). |
| Skeletal expansion using a miniscrew-assisted rapid palatal expansion in a 50-year-old patient | case report |
| Skeletal, Dentoalveolar and Dental Changes after "Mini-Screw Assisted Rapid Palatal Expansion" Evaluated with Cone Beam Computed Tomography | Patient population (< 13.5 years of age). |
| Stability of transversal correction with hybrid maxillary expansion appliance in bone and tegumental piriformis opening in relation to bone age and maturation of the midpalatal suture | Patient population (< 13.5 years of age). |
| The Hyrax appliance with tooth anchorage variations in surgically assisted rapid maxillary expansion: a finite element analysis | finite element analysis |
| The impact of mini-implant hybrid hyrax maxillary expansion on pulp blood flow and sensibility in healthy and traumatized teeth: A prospective study | No relevant results. |
| Three-dimensional assessment of asymmetric mid-palatal suture expansion assisted by a customized microimplant-supported rapid palatal expander in non-growing patients: Uncontrolled Clinical Trial | No relevant results. |
| Three-dimensional comparison of bone-borne and tooth-bone-borne maxillary expansion in young adults with maxillary skeletal deficiency | finite element analysis |
| What is the Oral Health-related Quality of Life following Miniscrew-Assisted Rapid Palatal Expansion (MARPE)? A prospective clinical cohort study | No relevant results. |
| Three-dimensional assessment of palatal area changes after posterior crossbite correction with tooth-borne and tooth bone-borne rapid maxillary expansion | No relevant results. |
