# Supplementary material for: Long-term efficacy and stability of miniscrew-assisted rapid palatal expansion in mid to late adolescents and adults: a systematic review and meta-analysis
Source: BMC Oral Health. 2023 Nov 3;23:829. doi: 10.1186/s12903-023-03574-y (PMC10623697; doi:10.1186/s12903-023-03574-y)
Supplement: Supplementary file 3 — Additional file 3: Supplementary Table 3. Results of individual studies for dental side effects at M1 by MARPE. [file 12903_2023_3574_MOESM3_ESM.docx]

**Supplementary Table 3.** Results of individual studies for dental side effects at M1 by MARPE. Measurement, mean ± SD (mm), 95% CI, range (mm), *p*-value and effect size were described when available.

| **Study** | **Measurement** | **Mean ± SD (mm)** | **95% CI lower/upper** | **Range (mm)** | ***P* value** | **Effect size** |
| --- | --- | --- | --- | --- | --- | --- |
| Li N et al. 2020 | The angle between the palatal root axis and nasal floor. | 4-all-bicortical: Lt 0.6 ± 0.5 Rt 0.8 ± 0.9 2-rear-bicortical: Lt 1.3 ± 1.1 Rt 1.4 ± 1.2 non-4-bicortical: Lt 4.7 ± 3.9 Rt 4.9 ± 3.3 |  |  | 4-all-bicortical: Lt 0.003 Rt 0.000 2-rear-bicortical: Lt 0.000 Rt 0.000 non-4-bicortical: Lt 0.001 Rt 0.000 |  |
| Lin et al. 2015 | The angle between the palatal root axis and the nasal floor. | Rt 1.16 ± 1.2 Lt 1.15 ± 1.05 |  |  | Rt 0.0022 Lt 0.0008 |  |
| Alsayegh et al. 2022 | The first molar angulation was determined by measuring the angle of intersection of the lines drawn tangent to the mesio-facial and mesio-palatal cusp tips of the maxillary first molars. | -1.9 ± 8.97 |  |  | NS |  |
| McMullen et al. 2022 | The angle between the long axis of the molars before and after treatment. | Rt 3.2 ± 2.9 Lt 3.5 ± 3.4 |  |  |  |  |
| Calil et al. 2021 | The angle between the line passing through the long axis of the tooth and vertical line parallel to the midsagittal plane. | Rt 4.14 ± 3.43 Lt 3.69 ± 2.90 |  |  |  |  |
| Lim et al. 2017 | The angle between the long axis of the M1 palatal root and the palatal plane. | 1.58 ± 4.61 |  |  | NS |  |

M1: first molar; Lt: left; Rt: right; NS: No Significant; CI: confidence interval; SD: standard deviation.
