# Supplementary material for: Long-term efficacy and stability of miniscrew-assisted rapid palatal expansion in mid to late adolescents and adults: a systematic review and meta-analysis
Source: BMC Oral Health. 2023 Nov 3;23:829. doi: 10.1186/s12903-023-03574-y (PMC10623697; doi:10.1186/s12903-023-03574-y)
Supplement: Supplementary file 4 — Additional file 4: Supplementary Table 4a. Results of individual studies for periodontal side effects (buccal inclination of alveolar bone at M1) by MARPE. Table 4b. Results of individual studies for periodontal side effects (buccal alveolar crest level at M1) by MARPE. [file 12903_2023_3574_MOESM4_ESM.docx]

**Supplementary Table 4a.** Results of individual studies for periodontal side effects (buccal inclination of alveolar bone at M1) by MARPE. Measurement, mean ± SD (mm), 95% CI, range (mm), *p*-value and effect size were described when available.

| **Study** | **Measurement** | **Mean ± SD (mm)** | **95% CI lower/upper** | **Range (mm)** | ***P* value** | **Effect size** |
| --- | --- | --- | --- | --- | --- | --- |
| Li N et al. 2020 | The angle between the palatal alveolar slope and nasal floor. | 4-all-bicortical: Lt 0.4 ± 0.5 Rt 0.6 ± 0.6 2-rear-bicortical: Lt 1.0 ± 0.8 Rt 1.1 ± 0.6 non-4-bicortical: Lt 2.1 ± 1.2 Rt 2.0 ± 1.3 |  |  | 4-all-bicortical: Lt 0.001 Rt 0.000 2-rear-bicortical: Lt 0.000 Rt 0.000 non-4-bicortical: Lt 0.000 Rt 0.000 |  |
| Lin et al. 2015 | The angle between the palatal alveolar slop and nasal floor. | Rt 1.43 ± 0.96 Lt 0.66 ± 0.66 |  |  | Rt 0.0001 Lt 0.0016 |  |
| Lim et al. 2017 | The angle between the palatal alveolar bone and the palatal plane. | 2.26 ± 4.85 |  |  | *p* < 0.05 |  |

M1: first molar; Lt: left; Rt: right; CI: confidence interval; SD: standard deviation.

**Table 4b.** Results of individual studies for periodontal side effects (buccal alveolar crest level at M1) by MARPE. Measurement, mean ± SD (mm), 95% CI, range (mm), *p*-value and effect size were described when available.

| **Study** | **Measurement** | **Mean ± SD (mm)** | **95% CI lower/upper** | **Range (mm)** | ***P* value** | **Effect size** |
| --- | --- | --- | --- | --- | --- | --- |
| Li N et al. 2020 | The alveolar crest change on the buccal side to the nasal floor before and after treatment. | 4-all-bicortical: Lt 0.5 ± 0.5 Rt 0.7 ± 0.6 2-rear-bicortical: Lt 0.7 ± 0.6 Rt 0.6 ± 0.3 non-4-bicortical: Lt 0.4 ± 0.9 Rt 0.8 ± 0.4 |  |  | 4-all-bicortical: Lt 0.000 Rt 0.000 2-rear-bicortical: Lt 0.000 Rt 0.000 non-4-bicortical: Lt 0.000 Rt 0.000 |  |
| Lin et al. 2015 | Alveolar bone dehiscence measured from the cement-enamel junction (CEJ) to the alveolar crest on buccal side. | Rt 0.11 ± 0.11 Lt 0.11 ± 0.12 |  |  | Rt 0.0025 Lt 0.0028 |  |
| Lim et al. 2017 | Distance from the cusp tip to the alveolar crest, along a reference line connecting the buccal cusp tip to the buccal root apex | 0.33 ± 0.67 |  |  | NS |  |

M1: first molar; Lt: left; Rt: right; NS: No Significant; CI: confidence interval; SD: standard deviation.

**Table 4c.** Results of individual studies for periodontal side effects (buccal alveolar bone thickness at M1) by MARPE. Measurement, mean ± SD (mm), 95% CI, range (mm), *p*-value and effect size were described when available.

| **Study** | **Measurement** | **Mean ± SD (mm)** | **95% CI lower/upper** | **Range (mm)** | ***P* value** | **Effect size** |
| --- | --- | --- | --- | --- | --- | --- |
| Calil et al. 2021 | Evaluated at the first molars in axial slices obtained 4 mm above the cementoenamel junction in the mesial of the maxillary right first molar. | Rt 0.24 ± 0.31 Lt 0.33 ± 0.35 |  |  | Rt 0.383  Lt 0.812 |  |
| Lim et al. 2017 | The shortest distances from the most prominent buccal root surface to external border of corresponding cortical bone. | 0.13 ± 0.59 |  |  | NS |  |

M1: first molar; Lt: left; Rt: right; NS: No Significant; CI: confidence interval; SD: standard deviation.
