# Supplementary material for: Long-term efficacy and stability of miniscrew-assisted rapid palatal expansion in mid to late adolescents and adults: a systematic review and meta-analysis
Source: BMC Oral Health. 2023 Nov 3;23:829. doi: 10.1186/s12903-023-03574-y (PMC10623697; doi:10.1186/s12903-023-03574-y)
Supplement: Supplementary file 5 — Additional file 5: Supplementary Table 5. Results of individual studies for craniofacial bone change by MARPE. [file 12903_2023_3574_MOESM5_ESM.docx]

**Supplementary Table 5.** Results of individual studies for craniofacial bone change by MARPE. Measurement, mean ± SD (mm), 95% CI, range (mm), *p*-value and effect size were described when available.

| **Study** | **Measurement** | **Mean ± SD (mm)** | **95% CI lower/upper** | **Range (mm)** | ***P* value** | **Effect size** |
| --- | --- | --- | --- | --- | --- | --- |
| An et al. 2021 | The distance between the left and right zygia (the most lateral aspect of the zygomatic arch) at posteroanterior cephalograms | 0.19 ± 0.63 |  |  | 0.211 |  |
| Li N et al. 2020 | The linear distance between the left and right lateral pterygoid plate measured at the axial slice crossing the palatal plane. | 4-all-bicortical: 1.7 ± 1.6 2-rear-bicortical: 1.3 ± 0.3 non-4-bicortical: 0.3 ± 0.3 |  |  | 4-all-bicortical: 0.000 2-rear-bicortical: 0.000 non-4-bicortical: 0.000 |  |
|  | The linear distance between the foramina of the left and right zygomatic bone measured at the axial slice. | 4-all-bicortical: 2.1 ± 0.8 2-rear-bicortical: 2.0 ± 0.7 non-4-bicortical: 1.1 ± 0.9 |  |  | 4-all-bicortical: 0.000 2-rear-bicortical: 0.000 non-4-bicortical: 0.000 |  |
|  | The linear distance between the left and right temporal bone measured at the axial slice crossing the inferior border of joint tubercle. | 4-all-bicortical: 0.6 ± 0.4 2-rear-bicortical: 0.5 ± 0.4 non-4-bicortical: 0.2 ± 0.3 |  |  | 4-all-bicortical: 0.000 2-rear-bicortical: 0.000 non-4-bicortical: 0.000 |  |
| McMullen et al. 2022 | Distance between right and left Or (Most inferior point of the orbital concavity in a frontal view, centered anterior-posteriorly on the orbital rim from the superior view). | 0.9 ± 0.7 |  |  | 0.06 |  |
|  | Distance between right and left zygomatic (The greatest point of convexity in which the horizontal and sagittal components of the zygomatic arch intersect in an inferior view). | 2.8 ± 1.8 |  |  | 0.4 |  |
|  | Distance between right and left palatine foramen (The most central point of the palatine foramen canal in anteroposterior direction in an inferior view at the palatal level). | 2.1 ± 1.3 |  |  | 0.022 |  |
| Tang et al. 2021 | Linear distance between the left and right lateral pterygoid plate measured at the axial slice crossing the palatal plane. | 1.26 ± 0.89 |  |  | ＜0.001 |  |
|  | Linear distance between the foramina of the left and right zygomatic bones measured at the axial slice; | 1.17 ± 0.95 |  |  | ＜0.001 |  |
|  | Linear distance between the left and right temporal bone measured at the axial slice crossing the inferior border of joint tubercle. | 1.08 ± 1.06 |  |  | ＜0.001 |  |
| Clement et al. 2017 | Comparison at pre‑ and post‑expansion: Lateral most border of frontonasal level. | 0.5±0.8366 |  |  | 0.1739 | 0.474342 |
|  | Comparison at pre‑ and post‑expansion: Lateral most border of zygoma. | 2.666±0.816 |  |  | 0.000* | 3.549648 |

CI: confidence interval; SD: standard deviation.
