# Supplementary material for: Long-term efficacy and stability of miniscrew-assisted rapid palatal expansion in mid to late adolescents and adults: a systematic review and meta-analysis
Source: BMC Oral Health. 2023 Nov 3;23:829. doi: 10.1186/s12903-023-03574-y (PMC10623697; doi:10.1186/s12903-023-03574-y)
Supplement: Supplementary file 6 — Additional file 6: Supplementary Table 6. Risk of bias assessment across studies according to the GRADE methodology. [file 12903_2023_3574_MOESM6_ESM.docx]

**Supplementary Table 6.** Risk of bias assessment across studies according to the GRADE methodology.

| Outcome | RISK OF BIAS | INCONSISTENCY | INDIRECTNESS | IMPRECISION | PUBLICAION BIAS | GRADE LEVEL OF EVIDENCE | Study |
| --- | --- | --- | --- | --- | --- | --- | --- |
| Maxillary basal bone width | Serious | Serious | Not serious | Serious | - | Very low | Li Q et al. 2020 Yi et al. 2020 Li N et al. 2020 Lin et al. 2015 Tang et al. 2021 |
| Maxillary alveolar bone width | Serious | Serious | Not serious | Not serious | Not serious | Very low | An et al. 2021 Yi et al. 2020 Li N et al. 2020 Lin et al. 2015 Calil et al. 2021 Tang et al. 2021 Lim et al. 2017 |
| Nasal floor width | Serious | Serious | Not serious | Not serious | Not serious | Very low | Li Q et al. 2020 Yi et al. 2020 Li N et al. 2020 Lin et al. 2015 Calil et al. 2021 Lim et al. 2017 |
| Nasal lateral width | Serious | Serious | Not serious | Serious | Not serious | Very low | Li Q et al. 2020 Yi et al. 2020 Li N et al. 2020 Tang et al. 2021 Lim et al. 2017 |
| ICW | Not serious | Serious | Not serious | Serious | - | Very low | Alsayegh et al. 2022  McMullen et al. 2022 Calil et al. 2021 Lim et al. 2017 Clement et al. 2017 |
| IP1W | Serious | Serious | Not serious | Serious | - | Very low | Yi et al. 2020 Lin et al. 2015 Calil et al. 2021 Lim et al. 2017 Clement et al. 2017 |
| IP2W | Serious | Serious | Not serious | Serious | - | Very low | Yi et al. 2020 Lin et al. 2015 Calil et al. 2021 Lim et al. 2017 Clement et al. 2017 |
| IMW | Serious | Serious | Not serious | Not serious | Not serious | Very low | An et al. 2021 Yi et al. 2020 Li N et al. 2020 Lin et al. 2015  Alsayegh et al. 2022 McMullen et al. 2022 Calil et al. 2021 Lim et al. 2017 Clement et al. 2017 |

ICW: intercanine width; IP1W: interpremolar width at P1; IP2W: interpremolar width at P2; IMW: intermolar width.
